# Selenium Exposure and Cancer Risk: an Updated Meta-analysis and Meta-regression

**DOI:** 10.1038/srep19213

**Published:** 2016-01-20

**Authors:** Xianlei Cai, Chen Wang, Wanqi Yu, Wenjie Fan, Shan Wang, Ning Shen, Pengcheng Wu, Xiuyang Li, Fudi Wang

**Affiliations:** 1 Institute of Environmental Medicine, Zhejiang University, P.R.China; 2 Ningbo Medical Treatment Center Lihuili Hospital, P.R.China; 3 Department of Clinic Medicine, Zhejiang University, Hangzhou, P.R.China; 4 Department of Epidemiology & Biostatistics, Zhejiang University, Hangzhou, P.R.China; 5 Department of Toxicology & Nutrition, Zhejiang University, Hangzhou, P.R.China

## Abstract

The objective of this study was to investigate the associations between selenium exposure and cancer risk. We identified 69 studies and applied meta-analysis, meta-regression and dose-response analysis to obtain available evidence. The results indicated that high selenium exposure had a protective effect on cancer risk (pooled OR = 0.78; 95%CI: 0.73–0.83). The results of linear and nonlinear dose-response analysis indicated that high serum/plasma selenium and toenail selenium had the efficacy on cancer prevention. However, we did not find a protective efficacy of selenium supplement. High selenium exposure may have different effects on specific types of cancer. It decreased the risk of breast cancer, lung cancer, esophageal cancer, gastric cancer, and prostate cancer, but it was not associated with colorectal cancer, bladder cancer, and skin cancer.

Selenium (Se) is an essential trace element having considerable and particular functions for human health because it is genetically encoded for which incorporation into proteins, as the constitutive part of selenocysteine, the 21^st^ amino acid^1^. Most se-proteins have been shown to have a wide range of pleiotropic effects, ranging from antioxidant to anti-inflammatory effects^2^, particularly the families of glutathione peroxidases (GPxs) and thioredoxin reductases (TrxRs)^1^, but their precise mechanism are not understood absolutely currently. Despite the scarce knowledge of mechanism, a large number of laboratory and ecologic researches focused on the associations between selenium and human health have been completed, showing that Se is associated with several human diseases including cardiovascular disease^3^,^4^,^5^, central nervous system disease^6^, diabetes mellitus^7^,^8^,^9^,^10^, and cancer, but the results are inconsistent.

We can see worldwide debates on the relation between selenium and cancer risk. Observational studies and randomized controlled trials suggest different effects in human. A new meta-analysis^11^ published in Cochrane 2014 described the association between selenium and cancer prevention, and this article tended to analyze the effect of selenium supplement based on random controlled trials. There are other similar meta-analyses have been published, few of them established dose-response or beneficial range of selenium exposure associated with the risk reduction or determined the shape of dose-response curve to find whether it is a linear relation, saturation or U-shaped curve relation between selenium exposure level and cancer risk. On the other hand, numerous new studies have been reported in recent years, and we think it is meaningful to conduct an updated meta-analysis including different types of cancer to provide comprehensive evidence and clarify the shape of dose-response association between selenium status and cancer risk.

## Methods

### Search strategy

**Figure 1:**
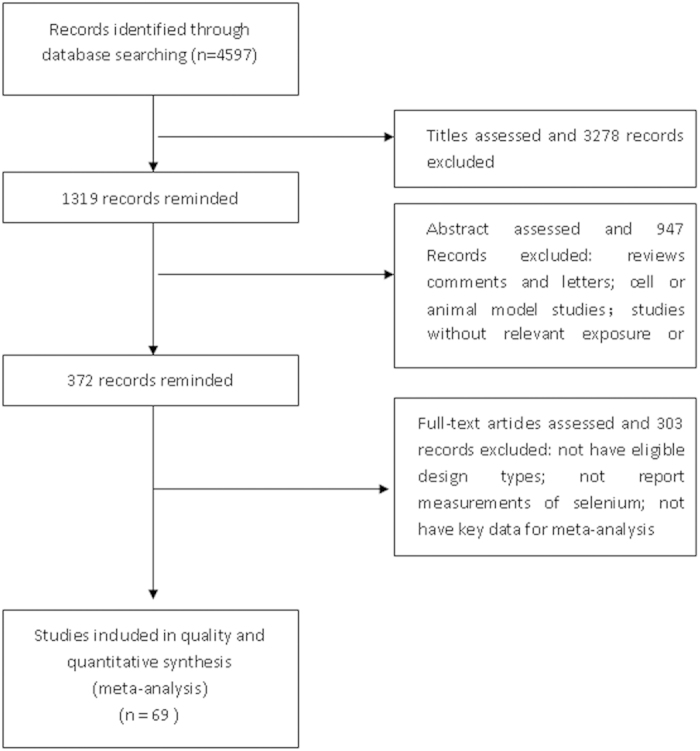
Flowchart of search strategy.

We carried out a systematic search for articles which described the relations between selenium and cancer risk in the medical and biologic databases (Medline 1980-March 2014, via Pubmed; Embase 1980-March 2014; Science Citation Index, Web of Science 1980- March 2014; CAB Health 1980- March 2014), using a comprehensive list of selenium/ selenium supplement/ serum/plasma selenium/ toenail selenium/ antioxidant/ minerals And cancer/ breast cancer/ lung cancer/ esophageal cancer/ gastric/stomach cancer/ colorectal cancer/ bladder cancer/ prostate cancer/skin cancer). We also searched references of relevant studies and reviews to identify works which were not found in the database search. The first two authors (Xianlei Cai and Chen Wang) conducted the search work (as shown in Fig. 1)

### Inclusion and exclusion criteria

Inclusion criteria were as follows: (1) was a randomized controlled trial, cohort or case-control study; (2) regarded selenium as baseline exposure, and cancer event (including incidence and mortality) as outcome; (3) were original works in English language which were published and indexed from January 1980 to March 2014; (4) had key date for meta-analysis or dose-response analysis.

Exclusion criteria were as follows: (1) was not involved with exposure-response associations between selenium and cancer risk; (2) cytological studies, animal studies, reviews, comments, abstracts and reviews; (3) low quality articles.

### Data extraction

All the data were extracted independently by three reviewers (Xianlei Cai, Chen Wang and Ning Shen) with a standardized data extraction form. The characteristics of the identified works were extracted as follows: first author name, year of publication, study country, design (RCT, cohort or case-control), number of subject (we extracted number of selenium exposure group and placebo group respectively from RCT studies, number of cohort participants from cohort studies, and number of case group plus control group from case-control studies), number of cases, age (mean or ranger), participants (men, women, both gender combined or special participants described in original studies), follow-up (year), Measurements of selenium (serum/plasma selenium, toenail selenium or selenium supplement), type of cancer, outcome, and estimates (odds ratio (OR), relative risk (RR) or hazard ratio (HR) at the highest compared with the lowest selenium exposure, with 95% confidence interval (CI)); Table 1 presents the summary data of each identified work in our meta-analysis.

### Quality assessment

We applied the Newcastle-Ottawa scale^12^,^13^ to assess the quality of the cohort and case-control studies. In this scale, one article was assessed on three perspectives: selection, comparability, outcome by using a “star system”. The maximum score was nine stars. We simply regarded scores of 0–3 stars as low quality, scores of 4–6 stars as moderate quality, and scores of 7–9 stars as high quality. According to RCTs, we used the Cochrane collaboration’s tool^14^ for assessing risk of bias from six domains: selection bias, performance bias, detection bias, attrition bias, reporting bias and other bias. Results were presented as low risk of bias, unclear risk of bias or high risk of bias.

### Statistical analyses

We extracted the multivariate-adjusted RRs, HRs or ORs and 95% confidence interval (CI) from original works. If some studies only provided 2×2 table data, we calculated the responding ORs. We considered these estimates as ORs when took those studies with different designs into account, for RRs and HRs were assumed to be the accurate estimates of ORs. Meta-regression analysis was conducted to figure out whether the associations between selenium exposure and cancer risk were influenced by some covariates (exposure modes, area and design), and we could recognize the influence factor with a positive meta-regression coefficient(*P* ≤ 0.05). We used Greenland and Longnecker^15^ method to conduct study-specific dose-response analyses based on the estimates of each category of plasma/serum selenium (ug/L), toenail selenium (ug/g) and selenium supplement (ug/d) respectively. We used mean or median of selenium exposure for each category when it was presented, and used midpoint when selenium exposure ranges were presented. When highest or lowest categories of selenium exposure were unbounded, we assumed the category width to be the same as the adjacent one. Number of subjects or person-time and number of cases for at least three categories of selenium exposure were also needed in dose-response analyses. We used restricted cubic splines method^16^ described by Orsini, N *et al.* to test the possible nonlinear relations, applying three fixed knots at 10%, 50% and 90% of selenium exposure level. The dose-response analyses were produced when there were more than 2 studies with relevant data.

Pooled ORs of selenium exposure with 95% confidence intervals (CI) for cancer risk were conducted by using fixed or random effects model. Heterogeneity was examined by using Q^17^ and *I*^*2*^ 18 index. When Q-test and I^2^ statistics does not presented a notable heterogeneity (*P* > 0.05 and *I*^*2*^ ≤ 50%), we used a fixed-effects analysis described by Mantel-Haenszel^19^. Otherwise, a random-effects analysis would be conducted described by DerSimonian and Laird method^20^. Publication bias was tested by Begger’s test and a weighted Egger test^21^,^22^. We also conducted sensitivity analyses by omitting one study at a time to present relative influence of each study on pooled ORs. Statistical calculations and figures were produced with software STATA version 12.0 (StataCorp LP, College Station, TX, USA).

## Results

### Characteristics of the study

The meta-analysis included 69 studies (26 case-control studies, 14 cohort studies, 19 nested case-control studies, 5 case-cohort studies, 5 randomized controlled trials) reporting 114 independent estimates (as shown in table 1) from Asia (4 studies from China, 2 from Japan, and 1 from Malaysia, Iran, and India, respectively), Europe (8 from Netherlands, 5 studies from Sweden, 5 from Finland, 3 from Poland, 2 from Belgium, 1 from Northern Ireland, Britain, Germany and France, respectively, and 3 studies from European countries) and America (27 studies from the United States, 2 from Canada and 1 from Austria). There were more than 364742 participants with 26138 cancer events. 5 studies used all types of cancer as outcome, 14 studies used breast cancer as outcome, 13 studies used lung cancer as outcome, 5 studies used esophageal cancer as outcome, 6 studies used gastric cancer as outcome, 10 studies used colorectal cancer as outcome, 9 studies used bladder cancer as outcome, 25 studies used prostate cancer as outcome, 4 studies used skin cancer as outcome, 1 study regarded urinary tract cancer, pancreas cancer, leukemia/lymphoma, uterine and ovarian cancer as outcome respectively. 11 studies^23^,^24^,^25^,^26^,^27^,^28^,^29^,^30^,^31^,^32^,^33^ mentioned above reported more than one cancer as an outcome, and several studies reported more than one estimate (as shown in table 1). 56 studies assessed biochemical selenium status: 37 used plasma/serum specimens and 19 used toenail specimens as exposure. 11 studies investigated selenium supplement or intake as exposure, using interviews or validated food frequency questionnaires. One study^34^ used breast tissue selenium as exposure, and the last one^35^ reported selenium intake, plasma selenium and toenail selenium as exposure respectively.

### Selenium exposure and all cancer

The relation between selenium exposure and all cancer risk, represented 114 independent estimates from 69 studies (as shown in Table 1). Meta-regression was done to detect the possible influencing factors, and we found that exposure mode (plasma/serum selenium, toenail selenium or selenium supplement), area (Asia, Europe and America) and design (case-control, cohort or RCT) were not influencing factors (exposure mode: *P* = 0.388; area: *P* = 0.523; design: *P* = 0.715). Therefore, we took the 114 estimates into meta-analysis. The result of the pooled analysis showed that high selenium exposure had a protective efficacy on cancer at the highest compared with the lowest category (pooled OR = 0.78; 95%CI: 0.73–0.83), with obvious heterogeneity (Q = 423.52; *P* = 0.000; I^2^ % = 73.3) and publication bias (Begger’s test *z*_*c*_ = 2.55, *P* = 0.011; Egger’s test *t* = −2.61, *P* = 0.010). Sensitivity analysis showed that the result was robust (as shown in Supplementary Table S1). The heterogeneity was due to a large amount of included estimates and different types of cancer.

**Figure 2:**
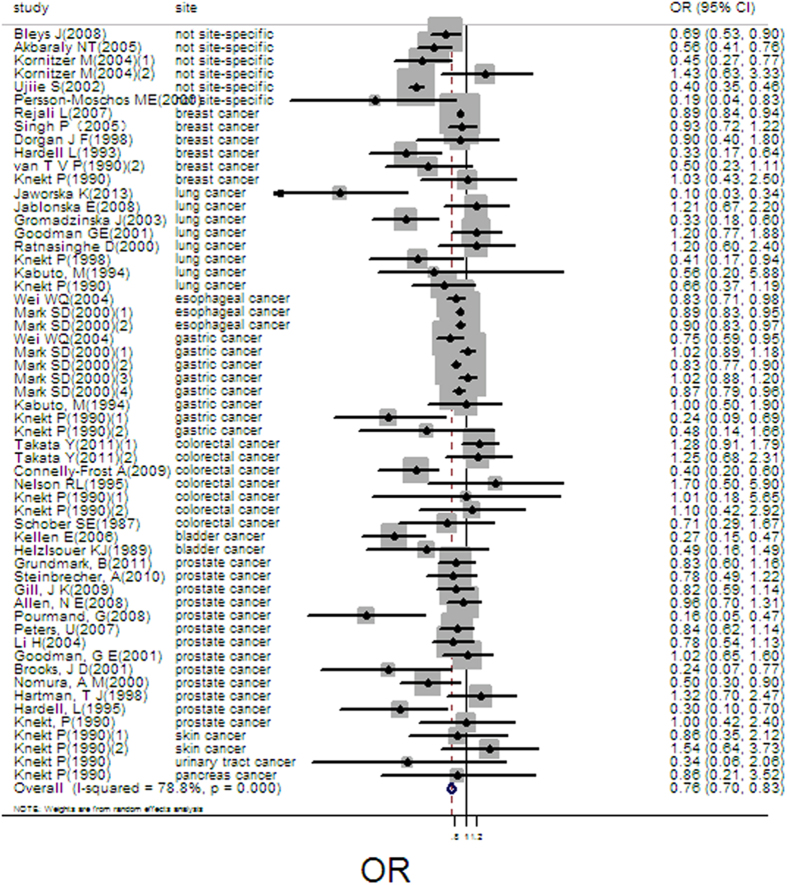
Forest plot of meta-analysis on serum/plasma selenium and cancer risk.

**Figure 3:**
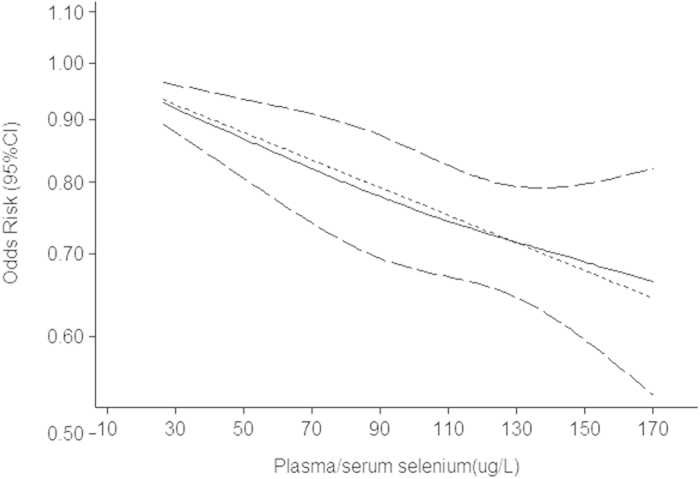
Summary nonlinear dose-response curves: plasma/serum selenium and cancer risk.

The pooled result from 58 independent estimates showed that high serum/plasma selenium had a effect on cancer prevention at the highest compared with the lowest category (pooled OR = 0.75, 95%CI: 0.69–0.82, Fig. 2), with obvious heterogeneity (Q = 268.57; *P* = 0.000; I^2^ % = 78.8) and publication bias (Begger’s test *z*_*c*_ = 2.54, *P* = 0.025; Egger’s test t = −2.43, *P* = 0.018). But the funnel plot was symmetry (supplementary Fig. S1). The heterogeneity could be due to a large amount of included estimates and publication bias. 17 groups of data were incorporated into dose-response analysis. The pooled OR was 0.95 (95%CI: 0.94–0.98) with 10 ug/L increase of plasma/serum selenium. Otherwise, we found obvious downward trends in the plots between plasma/serum selenium and total cancer risk in nonlinear dose-response analysis (*P* = 0.67 for non-linearity, Fig. 3).

**Figure 4:**
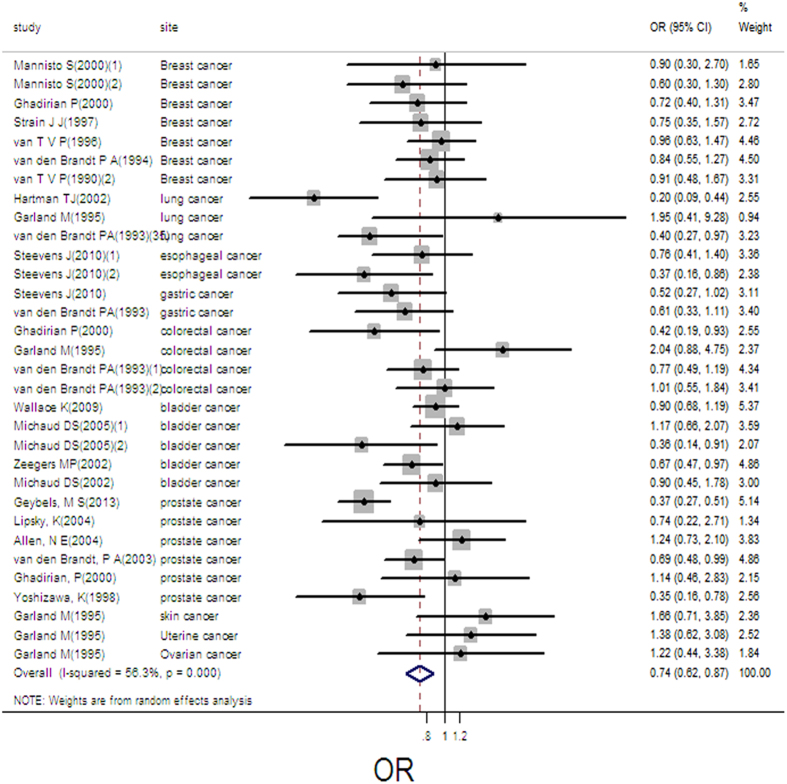
Forest plot of meta-analysis on toenail selenium and cancer risk.

**Figure 5:**
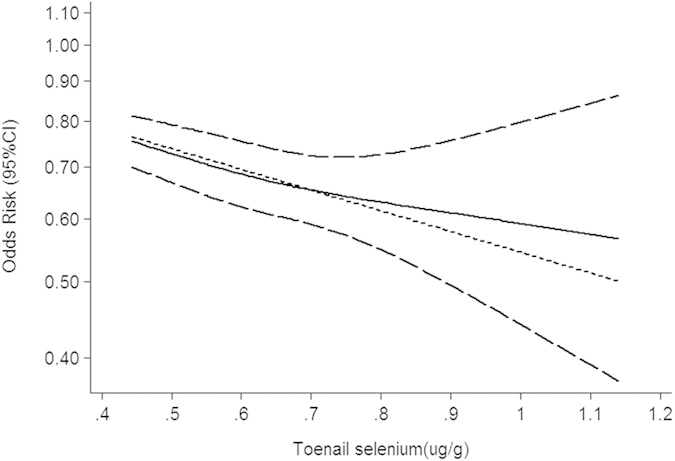
Summary nonlinear dose-response curves: toenail selenium and cancer risk.

There were 32 independent estimates describing the relation between toenail selenium and cancer risk. The result showed that high toenail selenium decreased cancer risk (pooled OR = 0.74, 95%CI: 0.62–0.87, as shown in Fig. 4), with obvious heterogeneity (Q = 70.95, *P* = 0.000; *I*^*2*^ % = 56.3). There was no publication bias (Begger’s test *z*_*c*_ = 0.05; *P* = 0.961; Egger’s test *t* = 0.52, *P* = 0.605), and the funnel plot did not show asymmetry (Fig. S2). 15 groups of data were incorporated into dose-response analysis. The pooled OR was 0.94 (95%CI: 0.92–0.97) with per 0.1 ug/g increase of toenail selenium. An downward trends was found in the plots of nonlinear dose-response analysis between toenail selenium and cancer risk (*P* = 0.500 for non-linearity, Fig. 5).

**Figure 6:**
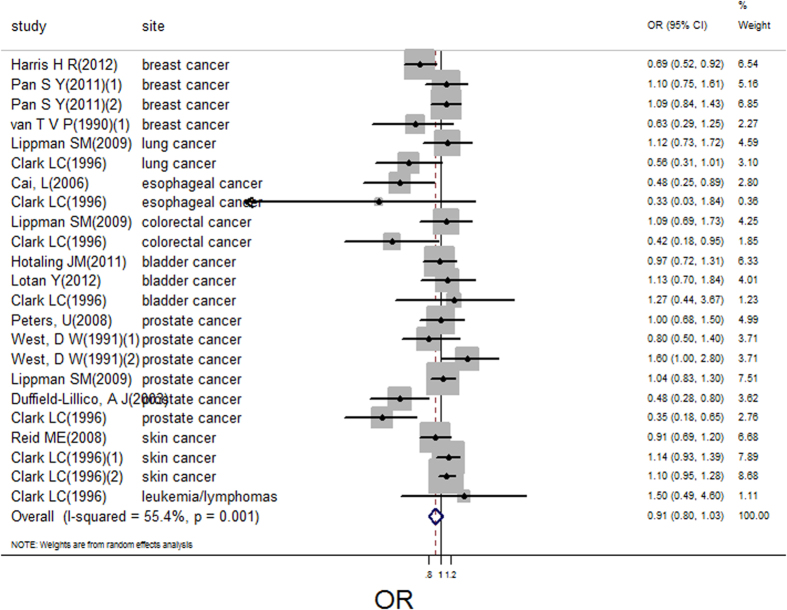
Forest plot of meta-analysis on selenium supplement and cancer risk.

There were 23 independent estimates describing the relation between selenium supplement and cancer risk. The result showed that selenium supplement was not associated with cancer risk (pooled OR = 0.91; 95%CI: 0.80–1.03, Fig. 6), with obvious heterogeneity (Q = 49.35, *P* = 0.001; *I*^*2*^ % = 55.4). There was no publication bias (Begger’s test *z*_*c*_ = 1.98; *P* = 0.05; Egger’s test *t* = 0.06, *P* = 0.21), and the funnel plot did not show asymmetry (Fig. S3). However, we just extracted two relevant data for selenium supplement and all cancer risk, the linear or nonlinear dose-response analysis was not conducted.

### Selenium exposure and breast cancer

**Figure 7:**
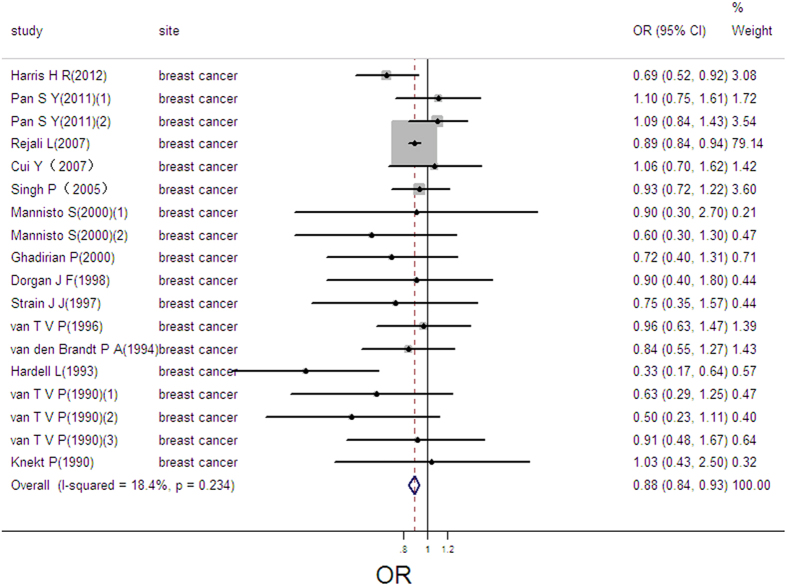
Forest plot of meta-analysis on selenium and breast cancer.

18 estimates from 14 studies were incorporated in the pooled analysis. We found that exposure mode, area and design were not influencing factor (exposure mode: *P* = 0.417; area: *P* = 0.705; design: *P* = 0.095) after Meta-regression. The pooled result showed that high selenium exposure decreased risk of breast cancer (pooled OR = 0.88; 95%CI: 0.84–0.93, Fig. 7), with no heterogeneity (Q = 20.83, *P* = 0.234; *I*^*2*^ % = 18.4) and publication bias (Begger’s test *z*_*c*_ = 1.74; *P* = 0.081; Egger’s test *t* = −1.21, *P* = 0.245). Sensitivity analysis showed the result was robust (as shown in Supplemental Table S1). We lacked sufficient data to conduct the linear or nonlinear dose-response analysis.

### Selenium exposure and lung cancer

**Figure 8:**
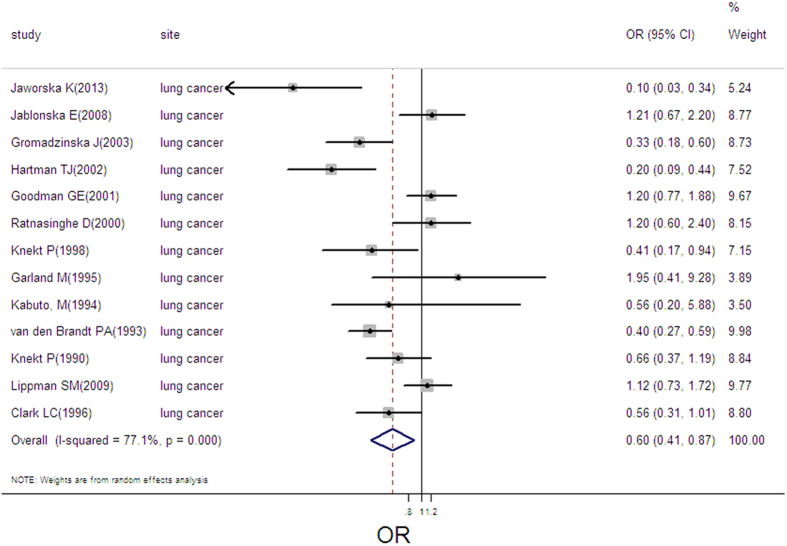
Forest plot of meta-analysis on selenium and lung cancer.

13 estimates from 13 studies were incorporated into the pooled analysis. We found that exposure mode, area and design were not influencing factor(exposure mode: *P* = 0.706; area: *P* = 0.581; design: *P* = 0.705). Therefore, we took the 13 estimates into meta-analysis. The result showed that high selenium exposure presented a protective effect on lung cancer (pooled OR = 0.60, 95%CI: 0.41–0.88, Fig. 8), with moderate heterogeneity (Q = 52.34, *P* = 0.000; *I*^*2*^ % = 77.1), but without publication bias (Begger’s test *z*_*c*_ = 1.16; *P* = 0.246; Egger’s test *t* = −0.79, *P* = 0.448). Sensitivity analysis showed the result was robust (Supplemental Table S1). 5 groups of data were incorporated into dose-response analysis between plasma/serum selenium and lung cancer risk. The result of linear dose-response analysis presented that plasma/serum selenium was not associated with cancer risk per 10 ug/L increases of plasma/serum selenium (pooled OR, 0.92; 95%CI: 0.83–1.01, *P* = 0.0001). Otherwise, we did not find a threshold effect in the plot between plasma/serum selenium and lung cancer risk in nonlinear dose-response analysis (*P* = 0.182 for non-linearity; Fig. S4).

### Selenium exposure and esophageal cancer

**Figure 9:**
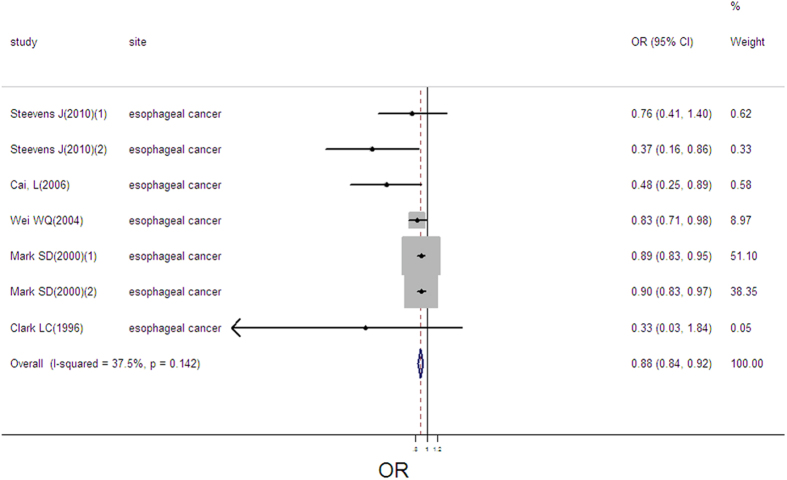
Forest plot of meta-analysis on selenium and esophageal cancer.

7 estimates from 5 studies were incorporated into the pooled analysis. The pooled OR was 0.88 (95%CI: 0.84–0.93, Fig. 9) with no heterogeneity (Q = 9.60, *P* = 0.142; *I*^*2*^ % = 37.5) and publication bias (Begger’s test *z*_*c*_ = 1.80; *P* = 0.072; Egger’s test *t* = −4.57, *P* = 0.006). Sensitivity analysis showed that the result was robust (Supplemental Table S1). We lacked sufficient data to conduct the linear or nonlinear dose-response analysis.

### Selenium exposure and gastric cancer

**Figure 10:**
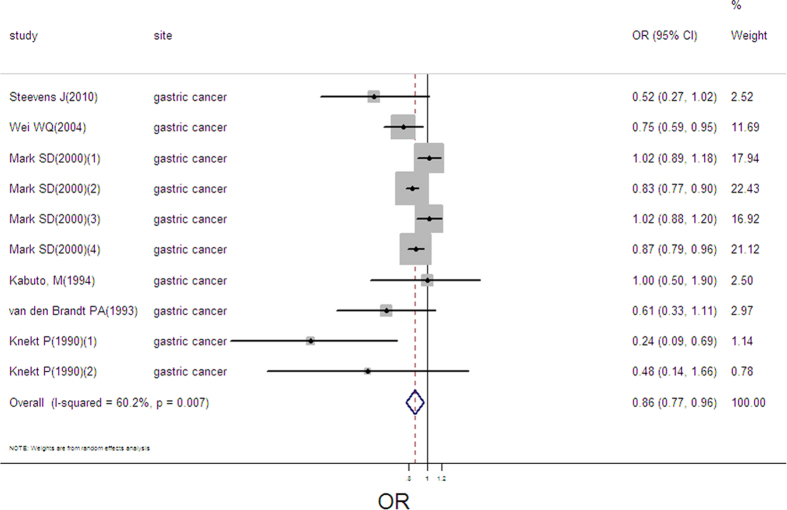
Forest plot of meta-analysis on selenium and gastric cancer.

10 estimates from 6 studies were incorporated into the pooled analysis. We found that exposure mode, area and design were not influencing factor (exposure mode: *P* = 0.288; area: *P* = 0.077; design: *P* = 0.769). Therefore, we took the 10 estimates into meta-analysis. The pooled OR was 0.86 (95%CI: 0.77–0.96, as shown in Fig. 10) with moderate heterogeneity (Q = 22.63, *P* = 0.007; *I*^*2*^ % = 60.2). There was no publication bias (Begger’s test *z*_*c*_ = 0.54; *P* = 0.592; Egger’s test *t* = −1.29, *P* = 0.235). Sensitivity analysis showed that the result was robust (as shown in Supplemental Table S1). We lacked sufficient data to conduct the linear or nonlinear dose-response analysis.

### Selenium exposure and colorectal cancer

**Figure 11:**
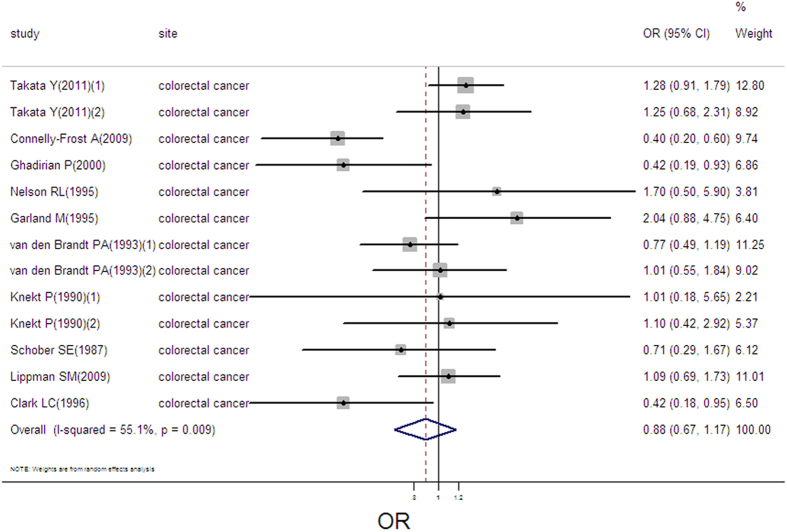
Forest plot of meta-analysis on selenium and colorectal cancer.

13 estimates from 10 studies were incorporated into the pooled analysis. We found that exposure mode, area and design were not influencing factor (exposure mode: *P* = 0.671; area: *P* = 0.871; design: *P* = 0.963). Therefore, we took the 13 estimates into meta-analysis. The result showed that high selenium exposure was not associated with colorectal cancer (pooled OR = 0.89, 95%CI: 0.67–1.17, Fig. 11), with moderate heterogeneity (Q = 26.71, *P* = 0.009; *I*^*2*^ % = 55.1), but without publication bias (Begger’s test *z*_*c*_ = 0.06; *P* = 0.951; Egger’s test *t* = −0.49, *P* = 0.634). Sensitivity analysis showed that the result was robust (Supplemental Table S1).

### Selenium exposure and bladder cancer

**Figure 12:**
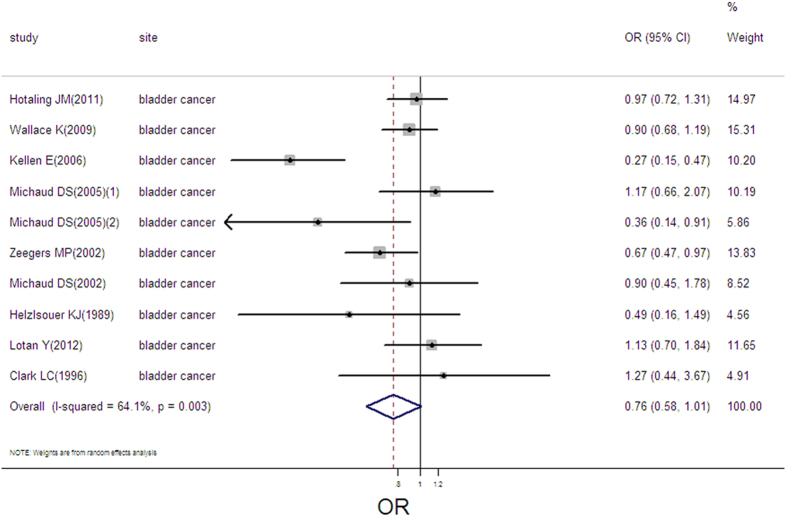
Forest plot of meta-analysis on selenium and bladder cancer.

10 estimates from 9 studies were incorporated in the pooled analysis. We found that exposure mode, area and design were not influencing factor (exposure mode: *P* = 0.05; area: *P* = 0.708; design: *P* = 0.601). Therefore, we took the 10 estimates into meta-analysis. The result showed that high selenium exposure was not associated with bladder cancer (pooled OR = 0.76, 95%CI: 0.58–1.01, as shown in Fig. 12) with moderate heterogeneity (Q = 25.06, *P* = 0.003; *I*^*2*^ % = 64.1), but without publication bias (Begger’s test *z*_*c*_ = 0.72; *P* = 0.474; Egger’s test *t* = −0.90, *P* = 0.395). 3 groups of data were incorporated into dose-response analysis between toenail selenium and bladder cancer risk. The consequence of linear dose-response analysis presented that toenail selenium was not associated with bladder cancer risk per 0.1 ug/g increase of toenail selenium (pooled OR = 0.95, 95%CI: 0.90–1.01). Otherwise, we did not find a threshold effect in the plot between toenail selenium and bladder cancer risk in nonlinear dose-response analysis (*P* = 0.413 for non-linearity; Fig. S5)

### Selenium exposure and prostate cancer

**Figure 13:**
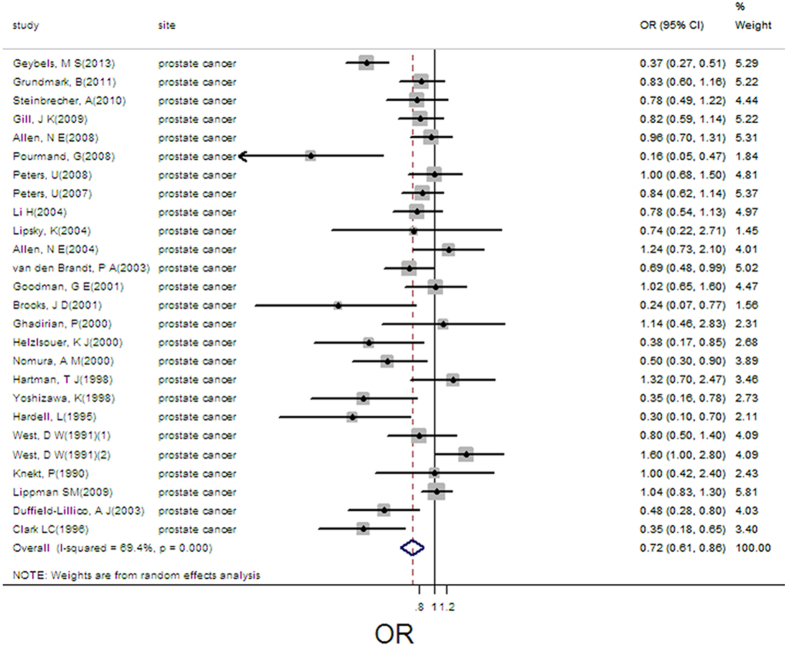
Forest plot of meta-analysis on selenium and prostate cancer.

26 estimates from 25 studies described the association between selenium and risk of prostate cancer. We found that exposure mode, area and design were not influencing factor (exposure mode: *P* = 0.682; area: *P* = 0.362; design: *P* = 0.478). Therefore, we took the 26 estimates into meta-analysis. The result showed that high selenium exposure decreased risk of prostate cancer (pooled OR = 0.72, 95%CI: 0.61–0.86, Fig. 13), with moderate heterogeneity (Q = 81.6, *P* = 0.000; *I*^*2*^ % = 69.4). There was no publication bias (Begger’s test *z*_*c*_ = 1.92; *P* = 0.055; Egger’s test *t* = −1.90, *P* = 0.070). Sensitivity analysis showed that the result was robust (Supplemental Table S1).

7 groups of data were incorporated into dose-response analysis between plasma/serum selenium and prostate cancer and 5 groups of data were included between toenail selenium and prostate cancer. The result of linear dose-response analysis presented that plasma/serum selenium was associated with prostate cancer risk per 10 ug/L increases (pooled OR = 0.97, 95%CI: 0.95–0.99; Q = 19.5, *P* = 0.003). The result presented that toenail selenium was associated with prostate cancer risk per 0.1 ug/g increases (pooled OR = 0.94, 95%CI: 0.89–0.99; Q = 20.27, *P* = 0.000). We did not find threshold effects in the plots between plasma/serum and toenail selenium and prostate cancer risk in nonlinear dose-response analyses (*P* = 0.739, *P* = 0.886 for non-linearity, respectively; Fig. S6,S7).

### Selenium exposure and risk of skin cancer

**Figure 14:**
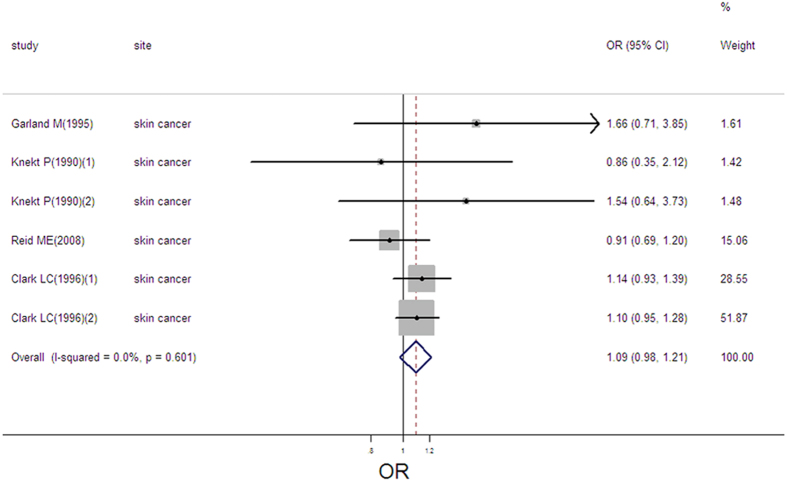
Forest plot of meta-analysis on selenium and skin cancer.

6 estimates from 4 studies were incorporated into the pooled analysis. We found that exposure mode and area were not influencing factor (exposure mode: *P* = 0.395; area: *P* = 0.454). Therefore, we took the 6 estimates into meta-analysis. The result of the pooled analysis showed that high selenium exposure was not associated with skin cancer (pooled OR = 1.09, 95%CI: 0.98–1.21, Fig. 14), with no heterogeneity (Q = 3.65, *P* = 0.601; *I*^*2*^ % = 0.0) and publication bias (Begger’s test *z*_*c*_ = 0.00; *P* = 1.000; Egger’s test *t* = 0.42, *P* = 0.697). Sensitivity analysis showed that the result was robust (Supplemental Table S1).

### Other subgroup analysis

The further stratified analysis were conducted by gender and study design. The results indicated that the protective effect of high selenium exposure had no gender difference (as shown in Table 2). When stratified by design, we found the results from observational studies presented the protective effect of selenium on cancer while the results from RCTs indicated null effect (as shown in Table 2).

## Discussion

Debating on Se-Cancer association is persistent. Selenium has been hypothesized to be a cancer preventive agent, a cancer therapeutic agent, or be a carcinogen^36^. Several^37^,^38^,^39^,^40^,^41^ studies presented results that blood selenium was associated with cancer. According to breast cancer, results from Harris H R *et al.*^42^, Rejali *et al.*^43^, and Hardell, L^44^ *et al.* studies presented a protective effect of selenium, while other observational studies^23^,^24^,^34^,^35^,^45^,^46^,^47^,^48^,^49^,^50^,^51^ showed null associations between selenium and breast cancer. For lung cancer, findings from Jaworska K *et al.*^52^, Gromadzinska, J *et al.*^53^, Hartman, T. J *et al.*^54^, Knekt, P. *et al.*^55^, van den Brandt, P. A *et al.*^56^ and Knekt, P *et al.*^24^ studies showed that high selenium exposure decreased lung cancer risk, but other 6 observational studies^24^,^25^,^26^,^33^,^57^,^58^ did not present similar results. Two randomized controlled trials^27^,^28^ found that selenium supplement was not associated with lung cancer (HR:1.12; 95%CI: 0.73–1.72; 0.56; 95%CI: 0.31–1.01, respectively). Several studies^23^,^24^,^25^,^26^,^28^,^29^,^30^,^31^,^32^,^59^,^60^,^61^,^62^,^63^ described the relation between digest system cancer, but the results were also inconsistent. Stevens, J *et al.*^29^ study presented that toenail selenium was associated with esophageal squamous cell carcinoma, but not with gastric cardia cancer. Wei WQ *et al.*^30^ study in China showed that serum selenium was associated with mortality of esophageal squamous cell carcinoma and gastric cardia cancer. Several studies^32^,^60^ presented null relation between serum selenium and colon cancer, rectal cancer. However, Clark LC *et al.*^28^’ randomized controlled trial showed selenium supplement decreased risk of colorectal cancer in people with skin carcinoma. According to bladder cancer, different studies^64^,^65^,^66^,^67^,^68^,^69^,^70^,^71^ showed different results. Hotaling JM *et al.*^64^ study presented that long-term use of supplemental selenium could not decrease bladder cancer risk after 6 years’ follow-up. Lotan Y *et al.*^71^ randomized controlled trial presented a similar result. Michaud, D. S *et al.*^67^ study showed a gender-specific relation between toenail selenium and bladder cancer that high toenail selenium had a protective effect on female bladder cancer. According to prostate cancer, the US Selenium and Vitamin E Cancer Prevention Trial showed that a long term oral supplement of selenomethionie(200ug/d) did not prevent prostate cancer^27^. And numerous observational studies^23^,^24^,^72^,^73^,^74^,^75^,^76^,^77^,^78^,^79^,^80^,^81^,^82^,^83^,^84^,^85^,^86^,^87^,^88^,^89^,^90^,^91^ also presented inconsistent results. Hurst, R *et al.*^92^ meta-analysis which included twelve studies showed that prostate cancer risk reduced with the increase of plasma/serum and toenail selenium. The Nutritional Prevention of Cancer Trial (NPCT)^28^ investigated the effect of selenium supplement on the development of skin cancer, and found no protective efficacy, Reid, M. E *et al.*^93^ study which was a sub-study of NPCT showed a similar result.

The results of meta-analysis suggest an inverse relation between selenium exposure and the total cancer risk (including breast cancer, lung cancer, esophageal cancer, gastric cancer, colorectal cancer, bladder cancer, prostate cancer, skin cancer, not site-specific cancer and other cancer). What is more, the results of dose-response analysis show a downward trend between plasma/serum selenium, toenail selenium and total cancer risk. The biomarker of selenium (serum/plasma and toenail selenium) was associated with cancer risk and could be easily measured and monitored to evaluate people health status. However, our results find that selenium supplement is not associated with cancer risk. Selenium supplement contains either inorganic or organic species or a mixture of both. The SELECT trial uses L-selenomethionie as an oral supplement, while the NPCT trial uses selenium yeast tablets. The different types of selenium supplement may present different effects on human health. On the other hand, first-pass elimination and bioavailability of different participants should be considered. Burk *et al.*^94^, study presents the results that the full expression of selenoprotein P requires more Se intake than that required by the full expression of GPxs, indicating that the Se intakes of the current studies are probably inadequate for optimizing the protective effects. We also cannot exclude the possibility that it is what associated with higher biochemical selenium level presents the shielding effect other than selenium exposure itself. We know that RCTs should research the association between selenium supplement and cancer risk, while observational studies usually research the relation between the biomarker of selenium and cancer risk. These reasons could be used to explain the inconsonant consequences of our stratified analysis by study design. And Vinceti, M^11^ *et al.*’ meta-analysis also find the inconsistent results between RCTs and observational studies. Future mechanism studies should pay more attention to the procedure from selenium supplement to biochemical selenium status to figure out the reasons for inconsonant effects of selenium supplement and biochemical selenium for preventing cancer. And future epidemiological studies and intervention trials should try to research selenium supplement, plasma/serum selenium and toenail selenium at the same time to reduce the potential bias.

We also find that selenium has diverse effects on specific types of cancer. According to breast cancer, we find an inverse relation when taking all relevant estimates into account. Nonetheless, we lack sufficient data to conduct dose-response analyses. According to lung cancer, we find that high selenium exposure presents a protective efficacy. Though a downward trend is seen in the nonlinear dose-response analysis, there is no statistical significance between plasma/serum selenium and lung cancer risk in linear dose-response analysis. The association between lung cancer and selenium exposure needs more discussion. According to esophageal cancer and gastric cancer, we find an obvious inverse relation. The quantity of estimates included in meta-analyses is not as many as other types of cancer, and we lack sufficient data to conduct dose-response analyses. According to colorectal cancer, we find no association between selenium exposure and cancer risk. Nevertheless, Ou Y *et al.*^95^ meta-analysis which included seven studies showed a protective effect of selenium on colorectal adenomas (OR = 0.67; 95%CI: 0.55–81). Selenium exposure probably plays a protective role in colorectal benign tumor rather than cancer, and the results need more researches. According to bladder cancer, we find no statistical significance between selenium exposure and bladder cancer. However, Amaral A F *et al.*^96^ meta-analysis which included seven epidemiologic studies presents that plasma/serum selenium and toenail selenium have protective effects on bladder cancer risk. According to prostate cancer, we find a protective effect of high selenium exposure for prostate cancer. The results of linear dose-response analyses between plasma/serum selenium, toenail selenium and prostate cancer support the result, and downward trends are shown in nonlinear dose-response analyses. However, two randomized controlled trials (the NPCT trial^28^ and the SELECT trial^27^) focusing on selenium supplement present the consequence that selenium supplement is not associated with prostate cancer risk. According to skin cancer, we find selenium is not associated with skin cancer risk.

There are numerous hypotheses about the potential anticarcinogenic mechanisms of selenium. The major positive effect may be contributed by the antioxidant function of GPxs and selenoprotein P^94^. Selenium is associated with the regulation of protein folding via the function of the endoplasmic reticulum to influence the process of necrosis and apoptosis of malignant cells^97^,^98^. Selenium also has the effect on DNA stability^98^. However, different malignant cells have their special biological characteristics and microenvironment for progress and invasion. They probably have disparate abilities of utilizing selenium. Hence, selenium probably has no effect on some types of cancer. The exact mechanism has yet to be investigated. On the other hand, the adverse effects of selenium supplement: mainly diabetes^27^,^99^, glaucoma^28^, and dermatologic alterations^27^ could not be ignored. So we should try to clarify what level of selenium supplement is needed for adequate nutrition and at what level dose is “unsafe”.

Our meta-analysis has several limitations clearly. Measurement errors in the assessment of selenium exposure may bias the effect estimates. Even among those studies regarding the same biochemical selenium as exposure, different measurement methods, different facilities and different staffs are all easy to produce measurement errors, and it is hard to make corrections. As showed in our inclusion criteria, we select case-control studies, cohort studies and RCTs into our meta-analysis. Selenium exposure may be linked to other behaviors like age, income, race, smoking status, alcohol consumption, body mass index, physical activity. These controlled confounding factors differ among sixty-nine studies and may influence the association between selenium exposure and cancer risk. Because of the insufficient number of relevant estimates, we have limited power to conduct subgroup analysis of pathological types of different cancer, and other controlled confounding factors.

Our study also has a few strength. We bring in a large number of studies and have largely avoided some main influencing factors by meta-regression analyses. And the robust outcomes of sensitivity analysis suggest that there is no distinct date making particularly contribution to the results. We detect the association between selenium exposure and different types of cancer to find a comprehensive understanding from global effects to local effects. We also conduct linear dose-response analyses which are stricter than high-versus-low analysis and the results of nonlinear dose-response analyses show dose-response trends in plots which are visual and accessible.

## Conclusions

High selenium exposure could decrease cancer risk, especially high plasma/serum selenium and toenail selenium. High selenium exposure may have dissimilar effects on specific types of cancer. Future epidemiological studies and intervention trials should try to research selenium supplement, plasma/serum selenium and toenail selenium at the same time to reduce the potential bias. The exact mechanism needs to be further investigated.

## Additional Information

**How to cite this article**: Cai, X. *et al.* Selenium Exposure and Cancer Risk: an Updated Meta-analysis and Meta-regression. *Sci. Rep.* **6**, 19213; doi: 10.1038/srep19213 (2016).

## Supplementary Material

supplementary table and figures

## Figures and Tables

**Table 1:** Characteristics of studies included in meta-analysis of studies on selenium and cancer.

Study	Country	Design	Subject	Case	age	Gender	Follow-up	Measurements of selenium	Type of cancer	OR(95%CI)	Quality sore
**Not site specific cancer**											
Bleys J(2008)	USA	cohort	13887	457	20–90	M and F	12 Y	Serum selenium	All cancer	0.69(0.53,0.90)	9
Akbaraly NT(2005)	France	cohort	1387	45	59–71	M and F	9 Y	Serum selenium	All cancer	0.56(0.41,0.76)	8
Kornitzer M(2004)	Belgium	nested case-control	539	139	25–74	Men	10 Y	Serum selenium	All cancer	0.45(0.27,0.77)	9
			195	50		Women				1.43(0.63,3.33)	
Ujiie S(2002)	Japan	cohort	5019	2707	N/A	M and F	5 Y	Serum selenium	All cancer	0.40(0.35,0.46)	7
Persson-Moschos ME(2000)	Sweden	nested case-control	903	302	middle age	Men	6 Y	Serum selenium	All cancer	0.19(0.04,0.83)	8
**Breast cancer**											
Harris H R(2012)	Swedish	cohort	66651	3146	mean 65	Women	9Y	Diet selenium	Breast cancer	0.69(0.52,0.92)	9
Pan S Y(2011)	Canada	case-control	4824	866	20–76	Premenopausal	N/A	Diet selenium	Breast cancer	1.10(0.75,1.61)	8
			4824	1496		Postmenopausal				1.09(0.84,1.43)	
Rejali L(2007)	Malaysia	matched case-control	124	62	mean 49	Women	N/A	Serum selenium	Breast cancer	0.89(0.84,0.94)	8
Cui Y (2007)	USA	Nested case-control	304	252	N/A	Women	N/A	Breast tissue selenium	Breast cancer	1.06(0.70,1.62)	9
Singh P (2005)	India	case-control	320	160	mean 45	Women	N/A	Serum selenium	Breast cancer	0.93(0.72,1.22)	8
Mannisto S(2000)	Finland	case-control	280	112	25–75	Premenopausal	N/A	Toenail	Breast cancer	0.90(0.30,2.70)	9
			442	177		Postmenopausal		selenium		0.60(0.30,1.30)	
Ghadirian P(2000)	Canada	case-control	1102	414	N/A	Women	N/A	Toenail selenium	Breast cancer	0.72(0.40,1.31)	8
Dorgan J F(1998)	USA	nested case-control	315	105	mean 58	Women	N/A	Serum selenium	Breast cancer	0.90(0.40,1.80)	9
Strain J J(1997)	Northern Ireland	case-control	204	99	mean 62	Postmenopausal	N/A	Toenail selenium	Breast cancer	0.75(0.35,1.57)	8
van T V P(1996)	Europe	case-control	605	266	50–74	Postmenopausal	N/A	Toenail selenium	Breast cancer	0.96(0.63,1.47)	8
van den Brandt P A(1994)	Netherlands	cohort	62537	355	55–69	Postmenopausal	3.3 Y	Toenail selenium	Breast cancer	0.84(0.55,1.27)	9
Hardell L(1993)	Sweden	case-control	632	441	20–84	Women	N/A	Serum selenium	Breast cancer	0.33(0.17,0.64)	7
van T V P(1990)	Netherlands	case-control	371	133	25–64	Women	N/A	Diet selenium	Breast cancer	0.63(0.29,1.25)	9
								Serum selenium		0.50(0.23,1.11)	
								Toenail selenium		0.91(0.48,1.67)	
Knekt P(1990)	Finland	cohort	N/A	48	15–99	women	N/A	Serum selenium	Breast cancer	1.03(0.43,2.50)	8
**Lung cancer**											
Jaworska K(2013)	Poland	case-control	172	86	mean 61.6	M and F	N/A	Serum selenium	Lung cancer	0.10(0.03,0.34)	8
Jablonska E(2008)	Poland	case-control	612	325	30–78	M and F	N/A	Serum selenium	Lung cancer	1.21(0.67,2.20)	8
Gromadzinska J(2003)	Poland	case-control	362	152	43–78	M and F	N/A	Serum selenium	Lung cancer	0.33(0.18,0.60)	8
Hartman TJ(2002)	Finland	Nested case-control	500	250	50–69	men	N/A	Toenail selenium	Lung cancer	0.20(0.09,0.44)	9
Goodman GE(2001)	USA	case-control	712	356	45–74	men	N/A	Serum selenium	Lung cancer	1.20(0.77,1.88)	9
Ratnasinghe D(2000)	China	nested case-control	324	108	35–74	men	6 Y	Serum selenium	Lung cancer	1.20(0.60,2.40)	9
Knekt P(1998)	Finland	nested case-control	285	95	mean 57	M and F	19 Y	Serum selenium	Lung cancer	0.41(0.17,0.94)	9
Garland M(1995)	USA	nested case-control	94	47	30–55	women	41 M	Toenail selenium	Lung cancer	1.95(0.41,9.28)	8
Kabuto, M(1994)	Japan	case-control	197	77	59–60	M and F	13 Y	Serum selenium	Lung cancer	0.56(0.20,5.88)	8
van den Brandt PA(1993)	Netherlands	cohort	3345	384	55–69	M and F	3.3 Y	Toenail selenium	Lung cancer	0.40(0.27,0.97)	9
Knekt P(1990)	Finland	cohort	N/A	153	15–99	men	N/A	Serum selenium	Lung cancer	0.66(0.37,1.19)	8
Lippman SM(2009)	USA, Canada, Puerto Rico	RCT	P:8696,e:8752	P:67,e: 75	≥50	men	5.46 Y	Selenium supplement	Lung cancer	1.12(0.73,1.72)	low risk of bias
Clark LC(1996)	USA	RCT	P:659, e: 653	P:35, e: 13	mean 63	M and F	6.4 Y	Selenium supplement	Lung cancer	0.56(0.31,1.01)	low risk of bias
**Esophageal cancer**											
Steevens J(2010)	Netherlands	case-cohort	3346	129	55–69	M and F	16.3 Y	Toenail selenium	EAC	0.76(0.41,1.40)	9
			3346	71					ESCC	0.37(0.16,0.86)	
Cai, L(2006)	China	case-cohort	633	218	N/A	M and F	10+ Y	Selenium intake	ESCC	0.48(0.25,0.89)	9
Wei WQ(2004)	China	cohort	1103	75	40–69	M and F	15 Y	Serum selenium	ESCC death	0.83(0.71,0.98)	9
Mark SD(2000)	China	case-cohort	1464	402	40–69	M and F	4.5 Y	Serum selenium	Incidence	0.89(0.83,0.95)	9
									morality	0.90(0.83,0.97)	
Clark LC(1996)	USA	RCT	P:659, e: 653	P:6, e: 2	mean 63	M and F	6.4 Y	Selenium supplement	esophageal cancer	0.33(0.03,1.84)	low risk of bias
**Gastric cancer**											
Steevens J(2010)	Netherlands	case-cohort	3346	114	55–69	M and F	16.3 Y	Toenail selenium	GCC	0.52(0.27,1.02)	9
Wei WQ(2004)	China	cohort	1103	36	40–69	M and F	15 Y	Serum selenium	GCC death	0.75(0.59,0.95)	9
Mark SD(2000)	China	case-cohort	1479	87	40–69	M and F	4.5 Y	Serum selenium	GNC onset	1.02(0.89,1.18)	9
			1652	590					GCC onset	0.83(0.77,0.90)	
			1149	87					GNC death	1.02(0.88,1.20)	
			1652	590					GCC death	0.87(0.79,0.96)	
Kabuto, M(1994)	Japan	case-control	428	202	59–60	M and F	13 Y	Serum selenium	gastric cancer	1.00(0.50,1.90)	8
van den Brandt PA(1993)	Netherlands	cohort	2459	92	55–69	M and F	3.3 Y	Toenail selenium	gastric cancer	0.61(0.33,1.11)	9
Knekt P(1990)	Finland	cohort	N/A	43	15–99	Men	N/A	Serum selenium	gastric cancer	0.24(0.09,0.69)	8
			N/A	30		Women				0.48(0.14,1.66)	
**Colorectal cancer**											
Takata Y(2011)	USA	nested case-control	1449	648	50–79	Women	N/A	Serum selenium	colon Ca	1.28(0.91,1.79)	9
			950	149					rectal Ca	1.25(0.68,2.31)	
Connelly-Frost A(2009)	USA	case-control	1362	532	40–80	M and F	N/A	Serum selenium	Colon cancer	0.40(0.20,0.60)	9
Ghadirian P(2000)	Canada	case-control	1090	402	N/A	M and F	N/A	Toenailselenium	colorectal cancer	0.42(0.19,0.93)	8
Nelson RL(1995)	USA	case-control	163	25	26–87	M and F	N/A	Serum selenium	colorectal cancer	1.70(0.50,5.90)	7
Garland M(1995)	USA	nested case-control	178	89	30–55	Women	41 M	Toenailselenium	colorectal cancer	2.04(0.88,4.75)	8
van den Brandt PA(1993)	Netherlands	cohort	2495	234	55–69	M and F	3.3 Y	Toenail	colon Ca	0.77(0.49,1.19)	9
			2495	113				selenium	rectal Ca	1.01(0.55,1.84)	
Knekt P(1990)	Finland	cohort	N/A	29	15–99	Men	N/A	Serum selenium	colorectal cancer	1.01(0.18,5.65)	8
				48		Women				1.10(0.42,2.92)	
Schober SE(1987)	USA	case-control	215	72	<75	M and F	N/A	Serum selenium	colon cancer	0.71(0.29,1.67)	7
Lippman SM(2009)	US, Canada, Puerto Rico	RCT	P:8696,e: 8752	P:60, e: 63	≥50	men	5.46 Y	Selenium supplement	colorectal cancer	1.09(0.69,1.73)	low risk of bias
Clark LC(1996)	USA	RCT	P:659, e: 653	P:19, e: 8	mean 63	M and F	6.4 Y	Selenium supplement	colorectal cancer	0.42(0.18,0.95)	low risk of bias
**Bladder cancer**											
Hotaling JM(2011)	USA	cohort	77050	330	50–76	M and F	6 Y	Selenium supplement	bladder cancer	0.97(0.72,1.31)	8
Wallace K(2009)	Germany	case-control	2048	857	25–74	M and F	N/A	Toenail selenium	bladder cancer	0.90(0.68,1.19)	9
Kellen E(2006)	Belgium	case-control	540	362	≥50	M and F	N/A	Serumselenium	bladder cancer	0.27(0.15,0.47)	9
Michaud DS(2005)	US	nested case-control	446	222	mean 62	Men	N/A	Toenail selenium	bladder cancer	1.17(0.66,2.07)	9
			233	116		Women				0.36(0.14,0.91)	
Zeegers MP(2002)	Netherlands	case-cohort	2890	431	55–69	M and F	6.3 Y	Toenail selenium	bladder cancer	0.67(0.47,0.97)	9
Michaud DS(2002)	Finland	nested case-control	264	132	50–69	M and F	N/A	Toenail selenium	bladder cancer	0.90(0.45,1.78)	8
Helzlsouer KJ(1989)	USA	case-control	95	35	mean 59	M and F	N/A	Serumselenium	bladder cancer	0.49(0.16,1.49)	9
Lotan Y(2012)	US, Canada, Puerto Rico	RCT	P:8696,e: 8752	P:35, e: 63	≥50	men	7.1 Y	Selenium supplement	bladder cancer	1.13(0.70,1.84)	low risk of bias
Clark LC(1996)	USA	RCT	P:659, e: 653	P:6, e: 8	mean 63	M and F	6.4 Y	Selenium supplement	bladder cancer	1.27(0.44,3.67)	low risk of bias
**Prostate cancer**											
Geybels, M S(2013)	Netherlands	Case-cohort	2074	898	55–69	Men	7 Y	Toenail selenium	prostate cancer	0.37(0.27,0.51)	9
Grundmark, B(2011)	Sweden	cohort	2045	208	50	Men	34 Y	Serum selenium	Prostate cancer	0.83(0.60,1.16)	9
Steinbrecher, A(2010)	European	Nested case-control	734	244	40–64	Men	N/A	Serum selenium	Prostate cancer	0.78(0.49,1.22)	9
Gill, J K(2009)	USA	Nested case-control	1403	467	45–75	Men	N/A	Serum selenium	Prostate cancer	0.82(0.59,1.14)	9
Allen, N E(2008)	Europe	Nested case-control	2018	959	43–76	Men	2.6–9.2 Y	Serum selenium	Prostate cancer	0.96(0.70,1.31)	9
Pourmand, G(2008)	Iran	case-control	130	62	40–90	Men	N/A	Serum selenium	Prostate cancer	0.16(0.06,0.47)	8
Peters, U(2008)	USA	cohort	35242	693	50–76	men	N/A	selenium supplement	Prostate cancer	1.00(0.68,1.50)	9
Peters, U(2007)	USA	Nested case-control	1603	724	55–74	men	8 Y	Serum selenium	Prostate cancer	0.84(0.62,1.14)	9
Lipsky, K(2004)	Austria	case-control	150	70	48–95	men	N/A	Toenail selenium	Prostate cancer	0.74(0.22,2.71)	8
Li H(2004)	USA	Nested case-control	1143	586	40–84	men	13 Y	Serum selenium	Prostate cancer	0.78(0.54,1.13)	9
Allen, N E(2004)	Britain	case-control	600	300	44–77	men	N/A	Toenail selenium	Prostate cancer	1.24(0.73,2.10)	9
van den Brandt, P A(2003)	Netherlands	Cohort	1751	540	55–69	men	6.3 Y	Toenail selenium	Prostate cancer	0.69(0.48,0.99)	9
Goodman, G E(2001)	USA	case-control	691	235	45–74	men	N/A	Serum selenium	Prostate cancer	1.02(0.65,1.60)	9
Brooks, J D(2001)	USA	case-control	148	52	68	men	N/A	Serum selenium	Prostate cancer	0.24(0.07,0.77)	9
Ghadirian, P(2000)	Canada	case-control	165	83	35–84	men	N/A	Toenail selenium	Prostate cancer	1.14(0.46,2.83)	8
Helzlsouer, K J(2000)	USA	Nested case-control	350	117	70	men	N/A	Serum selenium	Prostate cancer	0.38(0.17,0.85)	8
Nomura, A M(2000)	USA	Nested case-control	498	249	44–85	men	N/A	Serum selenium	Prostate cancer	0.50(0.30,0.90)	9
Hartman, T J(1998)	USA	cohort	29460	317	61	men	9 Y	Serum selenium	Prostate cancer	1.32(0.70,2.47)	9
Yoshizawa, K(1998)	USA	Nested case-control	362	181	40–75	men	7 Y	Toenail selenium	Prostate cancer	0.35(0.16,0.78)	9
Hardell, L(1995)	Sweden	case-control	245	124	44–87	men	N/A	Serum selenium	Prostate cancer	0.30(0.10,0.70)	7
West, D W(1991)	USA	case-control	564	179	45–67	men	N/A	selenium	Prostate cancer	0.80(0.50,1.40)	9
			473	179	68–74			supplement		1.60(1.00,2.80)	
Knekt, P(1990)	Finland	cohort	N/A	46	15–99	men	N/A	Serum selenium	Prostate cancer	1.00(0.42,2.4)	8
Lippman SM(2009)	US, Canada, Puerto Rico	RCT	P:8696,e: 8752	P:416	≥50	men	5.46 Y	Selenium supplement	Prostate cancer	1.04(0.83,1.30)	low risk of bias
				e:432							
Duffield-Lillico, A J(2003)	USA	RCT	P:470;	P: 42;	65	men	7.5 Y	selenium	Prostate cancer	0.48(0.28,0.80)	low risk of bias
			E:457	E: 22				supplement			
Clark LC(1996)	USA	RCT	P:659, e: 653	P:35, e: 13	mean 63	men	6.4 Y	Selenium supplement	Prostate cancer	0.35(0.18,0.65)	low risk of bias
**Skin cancer**											
Garland M(1995)	USA	nested case-control	30–55	126	63	women	41 M	Toenail selenium	melanoma	1.66(0.71,3.85)	8
Knekt P(1990)	USA	cohort	15–99	N/A	54	Men	N/A	Serum selenium	basal cell carcinoma	0.86(0.35,2.12)	8
					52	Women				1.54(0.64,3.75)	
Reid ME(2008)	USA	RCT	P:210, e: 213	P:108e: 98	mean 63	M and F	6.4 Y	Selenium supplement	non-melanoma skin cancer	0.91(0.69,1.20)	low risk of bias
Clark LC(1996)	USA	RCT	P:659, e: 653	P:190e:218	mean 63	M and F	6.4 Y	Selenium supplement	squamous cell carcinoma basal cell carcinoma	1.14(0.93,1.39)	low risk of bias
				P:350e:377						1.10(0.95,1.28)	
**Other cancer**											
Knekt P(1990)	USA	cohort	15–99	N/A	26	Men	N/A	Serum selenium	Urinary tract cancer	0.34(0.06,2.06)	8
Knekt P(1990)	USA	cohort	15–99	N/A	22	Women	N/A	Serum selenium	Pancreas cancer	0.86(0.21,3.52)	8
Clark LC(1996)	USA	RCT	P:659, e: 653	P:5, e: 8	mean 63	M and F	6.4 Y	Selenium supplement	leukemia/lymphomas	1.50(0.49,4.60)	low risk of bias
Garland M(1995)	USA	nestedcase-control	182	91	30–55	women	41 M	Toenail selenium	Uterine cancer	1.38(0.62,3.08)	8
Garland M(1995)	USA	nested case-control	182	91	30–55	women	41 M	Toenail selenium	Ovarian cancer	1.22(0.44,3.38)	8

Abbreviation: M and F: Male and Female; p: placebo; e: exposure; RCT: randomized controlled trials; N/A: not available; EAC: esophageal adenocarcinoma; ESCC: esophageal squamous cell carcinoma; GCC: gastric cardia cancer; GNC: gastric noncardia cancer; M: months; Y: years.

**Table 2:** The stratified analysis by gender and study design.

Subgroup	Type of subgroup	No of estimates	OR(95%CI)	Homogeneity test	*P*	I^2^(%)
				Q		
Design	cohort	40	0.75(0.68,0.82)	209.01	0.000	81.8
	Case-control	61	0.77(0.69,0.86)	162.63	0.000	63.7
	RCT	13	0.89(0.74,1.08)	31.32	0.002	61.7
Gender	Men	39	0.74(0.64,0.86)	111.94	0.000	66.1
	Women	31	0.90(0.86,0.95)	42.01	0.071	28.6
	Both combined	44	0.73(0.66,0.80)	260.02	0.000	83.5
