# Supplementary material for: Selenium Exposure and Cancer Risk: an Updated Meta-analysis and Meta-regression
Source: Sci Rep. 2016 Jan 20;6:19213. doi: 10.1038/srep19213 (PMC4726178; doi:10.1038/srep19213)

Selenium Exposure and Cancer Risk: an Updated Meta-analysis and Meta-regression

Xianlei Cai[1,2], Chen Wang[3], Wanqi Yu[4], Wenjie Fan[4], Shan Wang[4], Ning Shen[3], Pengcheng Wu[3] , Xiuyang Li[1,4*], Fudi Wang[5]

[1] Institute of Environmental Medicine, Zhejiang University, P.R.China

[2]Ningbo Medical Treatment Center Lihuili Hospital, P.R.China

[3] Department of Clinic Medicine, Zhejiang University, Hangzhou, P.R.China

[4] Department of Epidemiology & Biostatistics, Zhejiang University, Hangzhou, P.R.China

[5] Department of Toxicology & Nutrition, Zhejiang University, Hangzhou, P.R.China

The first two authors contributed equally to this work.

[*]Corresponding author:

Xiuyang Li

Email: lixiuyang@zju.edu.cn Tel:86-0571-88208192, Fax:86-0571-88208192

Department of Epidemiology & Biostatistics, Zhejiang University, 866Yuhangtang Road, 310058, Hangzhou, P.R.China

Supplementary Table 1. Sensitivity analyses of meta-analysis of the Se-cancer relation

	Site	OR(95%CI)	Q	p
**All cancer and selenium**				
Total	All cancer	0.78(0.73,0.83)	423.52	0.000
Bleys J(2008) excluded	not site-specific	0.782(0.732,0.835)	421.19	0.000
Akbaraly NT(2005) excluded	not site-specific	0.784(0.734,0.838)	416.56	0.000
Kornitzer M(2004)(1) excluded	not site-specific	0.784(0.735,0.838)	417.91	0.000
Kornitzer M(2004)(2) excluded	not site-specific	0.778(0.729,0.831)	422.00	0.000
Ujiie S(2002) excluded	not site-specific	0.801(0.756,0.850)	303.70	0.000
Persson-Moschos ME(2000) excluded	not site-specific	0.782(0.733,0.835)	419.79	0.000
Harris H R(2012) excluded	breast cancer	0.782(0.732,0.835)	421.51	0.000
Pan S Y(2011)(1) excluded	breast cancer	0.777(0.727,0.830)	421.72	0.000
Pan S Y(2011)(2) excluded	breast cancer	0.776(0.726,0.829)	420.05	0.000
Rejali L(2007) excluded	breast cancer	0.774(0.722,0.830)	419.89	0.000
Cui Y(2007) excluded	breast cancer	0.777(0.728,0.830)	422.42	0.000
Singh P(2005) excluded	breast cancer	0.778(0.728,0.831)	423.04	0.000
Mannisto S(2000)(1) excluded	breast cancer	0.780(0.730,0.833)	423.51	0.000
Mannisto S(2000)(2) excluded	breast cancer	0.781(0.732,0.834)	422.67	0.000
Ghadirian P(2000) excluded	breast cancer	0.781(0.731,0.834)	423.23	0.000
Dorgan J F(1998) excluded	breast cancer	0.779(0.730,0.833)	423.50	0.000
Strain J J(1997) excluded	breast cancer	0.780(0.731,0.833)	423.42	0.000
van T V P(1996) excluded	breast cancer	0.778(0.729,0.831)	423.19	0.000
van den Brandt P A(1994) excluded	breast cancer	0.779(0.730,0.833)	423.52	0.000
Hardell L(1993) excluded	breast cancer	0.785(0.735,0.838)	415.74	0.000
van T V P(1990)(1) excluded	breast cancer	0.781(0.731,0.834)	422.89	0.000
van T V P(1990)(2) excluded	breast cancer	0.782(0.732,0.835)	421.79	0.000
van T V P(1990)(3) excluded	breast cancer	0.779(0.730,0.832)	423.47	0.000
Knekt P(1990) excluded	breast cancer	0.779(0.730,0.832)	423.33	0.000
Jaworska K(2013) excluded	lung cancer	0.785(0.736,0.838)	411.61	0.000
Jablonska E(2008) excluded	lung cancer	0.778(0.728,0.830)	422.14	0.000
Gromadzinska J(2003) excluded	lung cancer	0.786(0.736,0.839)	414.08	0.000
Hartman TJ(2002) excluded	lung cancer	0.786(0.737,0.839)	410.79	0.000
Goodman GE(2001) excluded	lung cancer	0.777(0.727,0.830)	421.18	0.000
Ratnasinghe D(2000) excluded	lung cancer	0.778(0.729,0.831)	422.55	0.000
Knekt P(1998) excluded	lung cancer	0.783(0.733,0.836)	420.75	0.000
Garland M(1995) excluded	lung cancer	0.779(0.730,0.832)	422.42	0.000
Kabuto, M(1994) excluded	lung cancer	0.781(0.731,0.833)	423.29	0.000
van den Brandt PA(1993) excluded	lung cancer	0.787(0.738,0.840)	409.30	0.000
Knekt P(1990) excluded	lung cancer	0.781(0.731,0.834)	422.82	0.000
Lippman SM(2009) excluded	lung cancer	0.777(0.727,0.830)	421.89	0.000
Clark LC(1996) excluded	lung cancer	0.782(0.732,0.835)	421.63	0.000
Steevens J(2010)(1) excluded	esophageal cancer	0.780(0.731,0.833)	423.40	0.000
Steevens J(2010)(2) excluded	esophageal cancer	0.783(0.733,0.836)	419.79	0.000
Cai, L(2006) excluded	esophageal cancer	0.783(0.733,0.836)	420.44	0.000

Wei WQ(2004) excluded	esophageal cancer	0.779(0.728,0.832)	423.46	0.000
Mark SD(2000)(1) excluded	esophageal cancer	0.775(0.724,0.831)	421.17	0.000
Mark SD(2000)(2) excluded	esophageal cancer	0.776(0.725,0.831)	420.96	0.000
Clark LC(1996) excluded	esophageal cancer	0.781(0.731,0.834)	422.71	0.000
Steevens J(2010) excluded	gastric cancer	0.782(0.733,0.836)	421.44	0.000
Wei WQ(2004) excluded	gastric cancer	0.78(0.730,0.834)	422.50	0.000
Mark SD(2000)(1) excluded	gastric cancer	0.776(0.726,0.829)	416.66	0.000
Mark SD(2000)(2) excluded	gastric cancer	0.777(0.725,0.832)	423.22	0.000
Mark SD(2000)(3) excluded	gastric cancer	0.776(0.726,0.829)	417.88	0.000
Mark SD(2000)(4) excluded	gastric cancer	0.777(0.726,0.831)	423.22	0.000
Kabuto, M(1994) excluded	gastric cancer	0.779(0.729,0.832)	423.28	0.000
van den Brandt PA(1993) excluded	gastric cancer	0.782(0.732,0.835)	322.39	0.000
Knekt P(1990)(1) excluded	gastric cancer	0.784(0.734,0.837)	417.62	0.000
Knekt P(1990)(2) excluded	gastric cancer	0.781(0.732,0.834)	422.71	0.000
Takata Y(2011)(1) excluded	colorectal cancer	0.775(0.726,0.828)	417.78	0.000
Takata Y(2011)(2) excluded	colorectal cancer	0.777(0.728,0.830)	421.96	0.000
Connelly-Frost A(2009) excluded	colorectal cancer	0.785(0.735,0.838)	416.33	0.000
Ghadirian P(2000) excluded	colorectal cancer	0.783(0.733,0.836)	420.52	0.000
Nelson RL(1995) excluded	colorectal cancer	0.779(0.729,0.832)	422.30	0.000
Garland M(1995) excluded	colorectal cancer	0.777(0.728,0.829)	419.34	0.000
van den Brandt PA(1993)(1) excluded	colorectal cancer	0.780(0.730,0.833)	423.34	0.000
van den Brandt PA(1993)(2) excluded	colorectal cancer	0.779(0.729,0.832)	423.20	0.000
Knekt P(1990)(1) excluded	colorectal cancer	0.780(0.730,0.833)	423.48	0.000
Knekt P(1990)(2) excluded	colorectal cancer	0.779(0.730,0.832)	423.24	0.000
Schober SE(1987) excluded	colorectal cancer	0.780(0.731,0.834)	423.36	0.000
Lippman SM(2009) excluded	colorectal cancer	0.777(0.728,0.830)	422.36	0.000
Clark LC(1996) excluded	colorectal cancer	0.783(0.733,0.836)	420.78	0.000
Hotaling JM(2011) excluded	bladder cancer	0.778(0.728,0.831)	422.73	0.000
Wallace K(2009) excluded	bladder cancer	0.778(0.728,0.832)	423.34	0.000
Kellen E(2006) excluded	bladder cancer	0.788(0.738,0.841)	408.08	0.000
Michaud DS(2005)(1) excluded	bladder cancer	0.778(0.728,0.831)	422.29	0.000
Michaud DS(2005)(2) excluded	bladder cancer	0.783(0.733,0.836)	420.31	0.000
Zeegers MP(2002) excluded	bladder cancer	0.782(0.732,0.835)	421.90	0.000
Michaud DS(2002) excluded	bladder cancer	0.779(0.730,0.832)	423.49	0.000
Helzlsouer KJ(1989) excluded	bladder cancer	0.781(0.732,0.834)	422.59	0.000
Lotan Y(2012) excluded	bladder cancer	0.777(0.728,0.830)	422.15	0.000
Clark LC(1996) excluded	bladder cancer	0.779(0.729,0.832)	422.96	0.000
Geybels, M S(2013) excluded	prostate cancer	0.790(0.741,0.843)	397.28	0.000
Grundmark, B(2011) excluded	prostate cancer	0.779(0.729,0.833)	423.51	0.000
Steinbrecher, A(2010) excluded	prostate cancer	0.780(0.730,0.833)	423.39	0.000
Gill, J K(2009) excluded	prostate cancer	0.779(0.730,0.833)	423.48	0.000
Allen, N E(2008) excluded	prostate cancer	0.778(0.728,0.831)	422.91	0.000
Pourmand, G(2008) excluded	prostate cancer	0.785(0.735,0.837)	414.24	0.000
Peters, U(2008) excluded	prostate cancer	0.778(0.728,0.831)	422.84	0.000

Peters, U(2007) excluded	prostate cancer	0.779(0.729,0.832)	423.52	0.000
Li H(2004) excluded	prostate cancer	0.780(0.730,0.833)	423.33	0.000
Lipsky, K(2004) excluded	prostate cancer	0.780(0.731,0.833)	423.48	0.000
Allen, N E(2004) excluded	prostate cancer	0.777(0.728,0.830)	421.52	0.000
van den Brandt, P A(2003) excluded	prostate cancer	0.781(0.731,0.835)	422.28	0.000
Goodman, G E(2001) excluded	prostate cancer	0.778(0.728,0.831)	422.87	0.000
Brooks, J D(2001) excluded	prostate cancer	0.783(0.733,0.836)	419.27	0.000
Ghadirian, P(2000) excluded	prostate cancer	0.779(0.729,0.832)	423.11	0.000
Helzlsouer, K J(2000) excluded	prostate cancer	0.783(0.734,0.836)	419.70	0.000
Nomura, A M(2000) excluded	prostate cancer	0.783(0.733,0.836)	419.97	0.000
Hartman, T J(1998) excluded	prostate cancer	0.777(0.728,0.830)	421.62	0.000
Yoshizawa, K(1998) excluded	prostate cancer	0.784(0.734,0.837)	418.73	0.000
Hardell, L(1995) excluded	prostate cancer	0.783(0.734,0.836)	419.14	0.000
West, D W(1991)(1) excluded	prostate cancer	0.780(0.730,0.833)	423.47	0.000
West, D W(1991)(2) excluded	prostate cancer	0.775(0.726,0.828)	417.65	0.000
Knekt, P(1990) excluded	prostate cancer	0.779(0.730,0.832)	423.38	0.000
Lippman SM(2009) excluded	prostate cancer	0.776(0.727,0.829)	420.28	0.000
Duffield-Lillico, A J(2003) excluded	prostate cancer	0.784(0.734,0.837)	419.01	0.000
Clark LC(1996) excluded	prostate cancer	0.785(0.735,0.838)	416.22	0.000
Garland M(1995) excluded	skin cancer	0.778(0.728,0.830)	421.09	0.000
Knekt P(1990)(1) excluded	skin cancer	0.780(0.730,0.833)	423.52	0.000
Knekt P(1990)(2) excluded	skin cancer	0.778(0.728,0.831)	421.75	0.000
Reid ME(2008) excluded	skin cancer	0.778(0.728,0.831)	423.26	0.000
Clark LC(1996)(1) excluded	skin cancer	0.775(0.726,0.828)	415.02	0.000
Clark LC(1996)(2) excluded	skin cancer	0.775(0.726,0.828)	411.41	0.000
Knekt P(1990) excluded	urinary tract cancer	0.781(0.731,0.834)	422.50	0.000
Knekt P(1990) excluded	pancreas cancer	0.780(0.730,0.833)	423.52	0.000
Clark LC(1996) excluded	leukemia/lymphomas	0.779(0.729,0.832)	422.52	0.000
Garland M(1995) excluded	uterine cancer	0.778(0.728,0.831)	422.10	0.000
Garland M(1995) excluded	ovarian cancer	0.779(0.729,0.832)	423.03	0.000
**Not site-specific cancer and selenium**				
Total	not site-specific	0.55(0.40,0.76)	22.92	0.000
Bleys J(2008) excluded	not site-specific	0.507(0.357,0.722)	13.03	0.011
Akbaraly NT(2005) excluded	not site-specific	0.546(0.359,0.831)	21.46	0.000
Kornitzer M(2004)(1) excluded	not site-specific	0.569(0.389,0.832)	22.90	0.000
Kornitzer M(2004)(2) excluded	not site-specific	0.496(0.368,0.668)	15.90	0.003
Ujiie S(2002) excluded	not site-specific	0.620(0.517,0.743)	8.69	0.069
Persson-Moschos ME(2000) excluded	not site-specific	0.570(0.410,0.794)	21.56	0.000
**Breast cancer and selenium**				
Total	breast cancer	0.89(0.84,0.93)	20.83;	0.234
Harris H R(2012) excluded	breast cancer	0.892(0.848,0.938)	17.82	0.335
Pan S Y(2011)(1) excluded	breast cancer	0.882(0.838,0.927)	19.56	0.241
Pan S Y(2011)(2) excluded	breast cancer	0.878(0.835,0.924)	18.39	0.302
Rejali L(2007) excluded	breast cancer	0.866(0.776,0.966)	20.64	0.193

Cui Y(2007) excluded	breast cancer	0.883(0.839,0.928)	20.11	0.215
Singh P(2005) excluded	breast cancer	0.883(0.839,0.929)	20.69	0.191
Mannisto S(2000)(1) excluded	breast cancer	0.885(0.841,0.931)	19.62	0.187
Mannisto S(2000)(2) excluded	breast cancer	0.887(0.843,0.932)	19.75	0.232
Ghadirian P(2000) excluded	breast cancer	0.886(0.843,0.932)	20.36	0.204
Dorgan J F(1998) excluded	breast cancer	0.885(0.842,0.930)	20.83	0.185
Strain J J(1997) excluded	breast cancer	0.886(0.842,0.931)	20.64	0.193
van T V P(1996) excluded	breast cancer	0.884(0.840,0.930)	20.69	0.191
van den Brandt P A(1994) excluded	breast cancer	0.886(0.842,0.931)	20.77	0.188
Hardell L(1993) excluded	breast cancer	0.890(0.846,0.936)	12.27	0.725
van T V P(1990)(1) excluded	breast cancer	0.886(0.843,0.932)	20.00	0.220
van T V P(1990)(2) excluded	breast cancer	0.887(0.844,0.933)	18.80	0.279
van T V P(1990)(3) excluded	breast cancer	0.885(0.841,0.930)	20.82	0.185
Knekt P(1990) excluded	breast cancer	0.884(0.841,0.930)	20.72	0.190
**Lung cancer and selenium**				
Total	lung cancer	0.60(0.41,0.88)	52.34	0.000
Jaworska K(2013) excluded	lung cancer	0.660(0.459,0.948)	42.95	0.000
Jablonska E(2008) excluded	lung cancer	0.558(0.374,0.833)	47.89	0.000
Gromadzinska J(2003) excluded	lung cancer	0.633(0.425,0.941)	46.93	0.000
Hartman TJ(2002) excluded	lung cancer	0.656(0.451,0.952)	43.35	0.000
Goodman GE(2001) excluded	lung cancer	0.555(0.373,0.825)	44.09	0.000
Ratnasinghe D(2000) excluded	lung cancer	0.561(0.376,0.838)	49.23	0.000
Knekt P(1998) excluded	lung cancer	0.614(0.411,0.919)	51.14	0.000
Garland M(1995) excluded	lung cancer	0.570(0.386,0.840)	50.44	0.000
Kabuto, M(1994) excluded	lung cancer	0.598(0.403,0.887)	52.31	0.000
van den Brandt PA(1993) excluded	lung cancer	0.624(0.415,0.938)	44.81	0.000
Knekt P(1990) excluded	lung cancer	0.589(0.388,0.896)	52.34	0.000
Lippman SM(2009) excluded	lung cancer	0.558(0.372,0.836)	45.21	0.000
Clark LC(1996) excluded	lung cancer	0.599(0.394,0.909)	52.05	0.000
**Esophageal cancer and selenium**				
Total	esophageal cancer	0.88(0.84,0.93)	9.60	0.142
Steevens J(2010)(1) excluded	esophageal cancer	0.882(0.840,0.926)	9.38	0.095
Steevens J(2010)(2) excluded	esophageal cancer	0.884(0.842,0.927)	5.50	0.358
Cai, L(2006) excluded	esophageal cancer	0.884(0.843,0.928)	6.06	0.300
Wei WQ(2004) excluded	esophageal cancer	0.886(0.843,0.932)	9.02	0.108
Mark SD(2000)(1) excluded	esophageal cancer	0.872(0.814,0.934)	9.43	0.093
Mark SD(2000)(2) excluded	esophageal cancer	0.870(0.818,0.925)	9.14	0.103
Clark LC(1996) excluded	esophageal cancer	0.882(0.840,0.925)	8.73	0.120
**Gastric cancer and selenium**				
Total	gastric cancer	0.86(0.77,0.96)	22.63	0.007
Steevens J(2010) excluded	gastric cancer	0.873(0.782,0.973)	20.30	0.009
Wei WQ(2004) excluded	gastric cancer	0.875(0.776,0.986)	21.02	0.007
Mark SD(2000)(1) excluded	gastric cancer	0.832(0.741,0.935)	17.16	0.028
Mark SD(2000)(2) excluded	gastric cancer	0.852(0.736,0.987)	19.98	0.010

Mark SD(2000)(3) excluded	gastric cancer	0.833(0.740,0.937)	18.22	0.020
Mark SD(2000)(4) excluded	gastric cancer	0.839(0.721,0.975)	22.63	0.004
Kabuto, M(1994) excluded	gastric cancer	0.855(0.762,0.959)	22.47	0.004
van den Brandt PA(1993) excluded	gastric cancer	0.869(0.777,0.972)	21.29	0.006
Knekt P(1990)(1) excluded	gastric cancer	0.878(0.798,0.966)	16.45	0.036
Knekt P(1990)(2) excluded	gastric cancer	0.864(0.772,0.966)	21.74	0.005
**Colorectal cancer and selenium**				
Total	colorectal cancer	0.89(0.67,1.17)	26.71	0.009
Takata Y(2011)(1) excluded	colorectal cancer	0.838(0.624,1.125)	21.94	0.025
Takata Y(2011)(2) excluded	colorectal cancer	0.855(0.635,1.151)	25.69	0.007
Connelly-Frost A(2009) excluded	colorectal cancer	1.008(0.844,1.203)	16.87	0.112
Ghadirian P(2000) excluded	colorectal cancer	0.934(0.711,1.228)	22.75	0.019
Nelson RL(1995) excluded	colorectal cancer	0.862(0.649,1.145)	25.75	0.007
Garland M(1995) excluded	colorectal cancer	0.837(0.636,1.102)	23.17	0.017
van den Brandt PA(1993)(1) excluded	colorectal cancer	0.899(0.660,1.226)	25.95	0.007
van den Brandt PA(1993)(2) excluded	colorectal cancer	0.873(0.644,1.182)	26.62	0.005
Knekt P(1990)(1) excluded	colorectal cancer	0.882(0.663,1.173)	26.70	0.005
Knekt P(1990)(2) excluded	colorectal cancer	0.873(0.652,1.170)	26.58	0.005
Schober SE(1987) excluded	colorectal cancer	0.897(0.670,1.203)	26.35	0.006
Lippman SM(2009) excluded	colorectal cancer	0.862(0.633,1.175)	26.13	0.006
Clark LC(1996) excluded	colorectal cancer	0.931(0.708,1.226)	23.11	0.017
**Bladder cancer ad selenium**				
Total	bladder cancer	0.76(0.58,1.01)	25.06	0.003
Hotaling JM(2011) excluded	bladder cancer	0.729(0.527,1.008)	23.39	0.003
Wallace K(2009) excluded	bladder cancer	0.736(0.527,1.029)	24.42	0.002
Kellen E(2006) excluded	bladder cancer	0.882(0.759,1.026)	9.61	0.293
Michaud DS(2005)(1) excluded	bladder cancer	0.727(0.541,0.977)	23.43	0.003
Michaud DS(2005)(2) excluded	bladder cancer	0.802(0.609,1.055)	22.04	0.005
Zeegers MP(2002) excluded	bladder cancer	0.776(0.566,1.063)	23.69	0.003
Michaud DS(2002) excluded	bladder cancer	0.750(0.556,1.011)	24.98	0.002
Helzlsouer KJ(1989) excluded	bladder cancer	0.780(0.587,1.037)	24.24	0.002
Lotan Y(2012) excluded	bladder cancer	0.724(0.537,0.977)	23.15	0.003
Clark LC(1996) excluded	bladder cancer	0.743(0.558,0.990)	24.38	0.002
**Prostate cancer and selenium**				
Total	prostate cancer	0.72(0.61,0.86)	81.6	0.000
Geybels, M S(2013) excluded	prostate cancer	0.764(0.653,0.892)	58.75	0.000
Grundmark, B(2011) excluded	prostate cancer	0.715(0.596,0.858)	81.46	0.000
Steinbrecher, A(2010) excluded	prostate cancer	0.719(0.601,0.860)	81.60	0.000
Gill, J K(2009) excluded	prostate cancer	0.716(0.596,0.859)	81.51	0.000
Allen, N E(2008) excluded	prostate cancer	0.710(0.592,0.851)	79.78	0.000
Pourmand, G(2008) excluded	prostate cancer	0.746(0.632,0.881)	73.17	0.000
Peters, U(2008) excluded	prostate cancer	0.710(0.594,0.849)	80.01	0.000
Peters, U(2007) excluded	prostate cancer	0.714(0.595,0.858)	81.36	0.000
Li H(2004) excluded	prostate cancer	0.718(0.599,0.861)	81.60	0.000

Lipsky, K(2004) excluded	prostate cancer	0.722(0.607,0.860)	81.59	0.000
Allen, N E(2004) excluded	prostate cancer	0.707(0.593,0.843)	78.56	0.000
van den Brandt, P A(2003) excluded	prostate cancer	0.723(0.603,0.866)	81.13	0.000
Goodman, G E(2001) excluded	prostate cancer	0.711(0.595,0.849)	80.19	0.000
Brooks, J D(2001) excluded	prostate cancer	0.737(0.621,0.874)	77.87	0.000
Ghadirian, P(2000) excluded	prostate cancer	0.715(0.600,0.852)	80.93	0.000
Helzlsouer, K J(2000) excluded	prostate cancer	0.737(0.620,0.876)	78.50	0.000
Nomura, A M(2000) excluded	prostate cancer	0.734(0.616,0.875)	79.02	0.000
Hartman, T J(1998) excluded	prostate cancer	0.708(0.595,0.843)	78.88	0.000
Yoshizawa, K(1998) excluded	prostate cancer	0.739(0.622,0.878)	77.62	0.000
Hardell, L(1995) excluded	prostate cancer	0.738(0.622,0.876)	77.87	0.000
West, D W(1991)(1) excluded	prostate cancer	0.719(0.601,0.859)	81.59	0.000
West, D W(1991)(2) excluded	prostate cancer	0.702(0.592,0.832)	73.91	0.000
Knekt, P(1990) excluded	prostate cancer	0.717(0.601,0.854)	81.29	0.000
Lippman SM(2009) excluded	prostate cancer	0.706(0.590,0.845)	74.18	0.000
Duffield-Lillico, A J(2003) excluded	prostate cancer	0.736(0.618,0.877)	78.22	0.000
Clark LC(1996) excluded	prostate cancer	0.744(0.627,0.882)	75.50	0.000
**Skin cancer and selenium**				
Total	skin cancer	1.09(0.98,1.21)	3..65	0.601
Garland M(1995) excluded	skin cancer	1.081(0.970,1.205)	2.68	0.613
Knekt P(1990)(1) excluded	skin cancer	1.093(0.981,1.217)	3.38	0.496
Knekt P(1990)(2) excluded	skin cancer	1.083(0.972,1.207)	3.04	0.550
Reid ME(2008) excluded	skin cancer	1.124(1.000,1.263)	1.75	0.782
Clark LC(1996)(1) excluded	skin cancer	1.069(0.941,1.214)	3.37	0.498
Clark LC(1996)(2) excluded	skin cancer	1.077(0.922,1.257)	3.61	0.461

Supplementary Fig 1 Funnel plot of meta-analysis on serum/plasma selenium and cancer risk

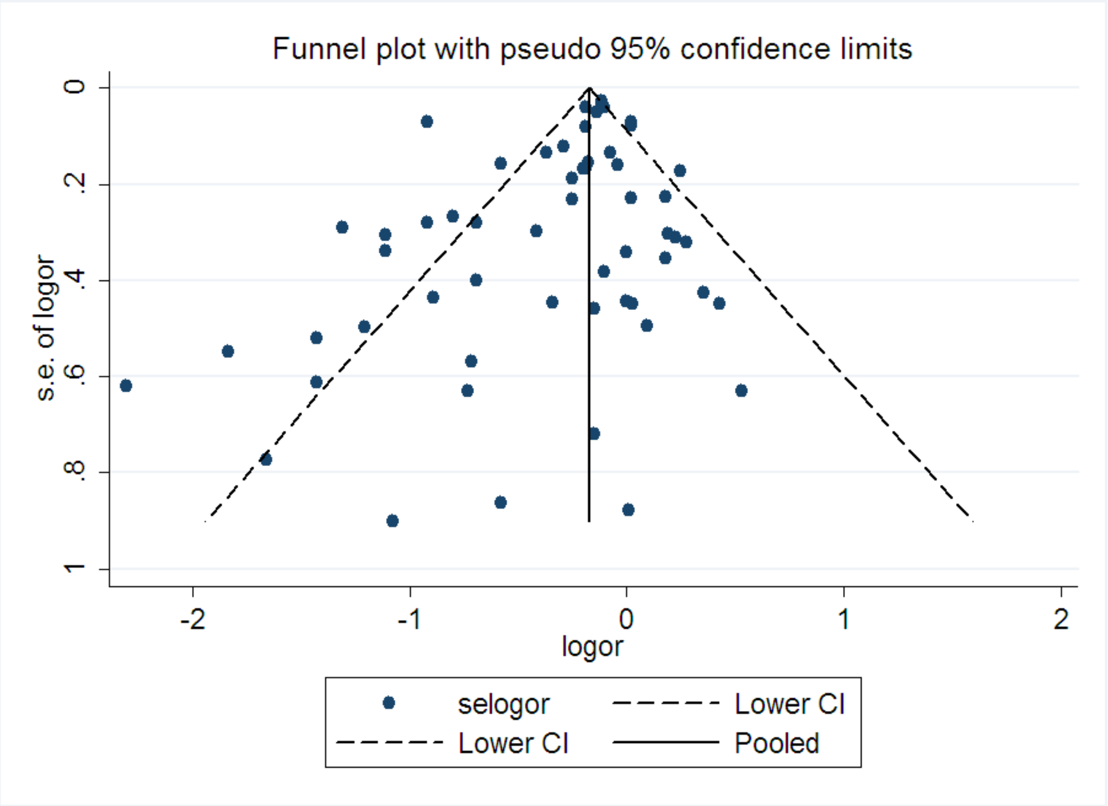

Supplementary Fig 2 Funnel plot of meta-analysis on toenail selenium and cancer risk

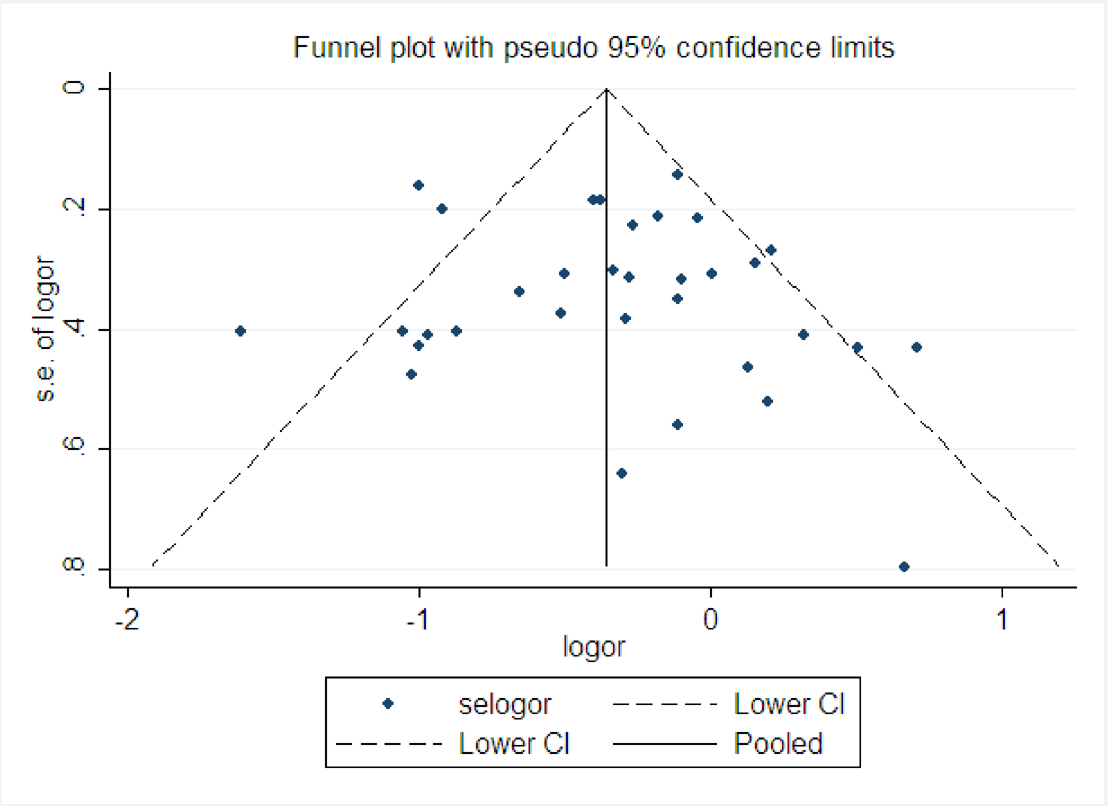

Supplementary Fig 3 Funnel plot of meta-analysis on selenium supplement and cancer risk

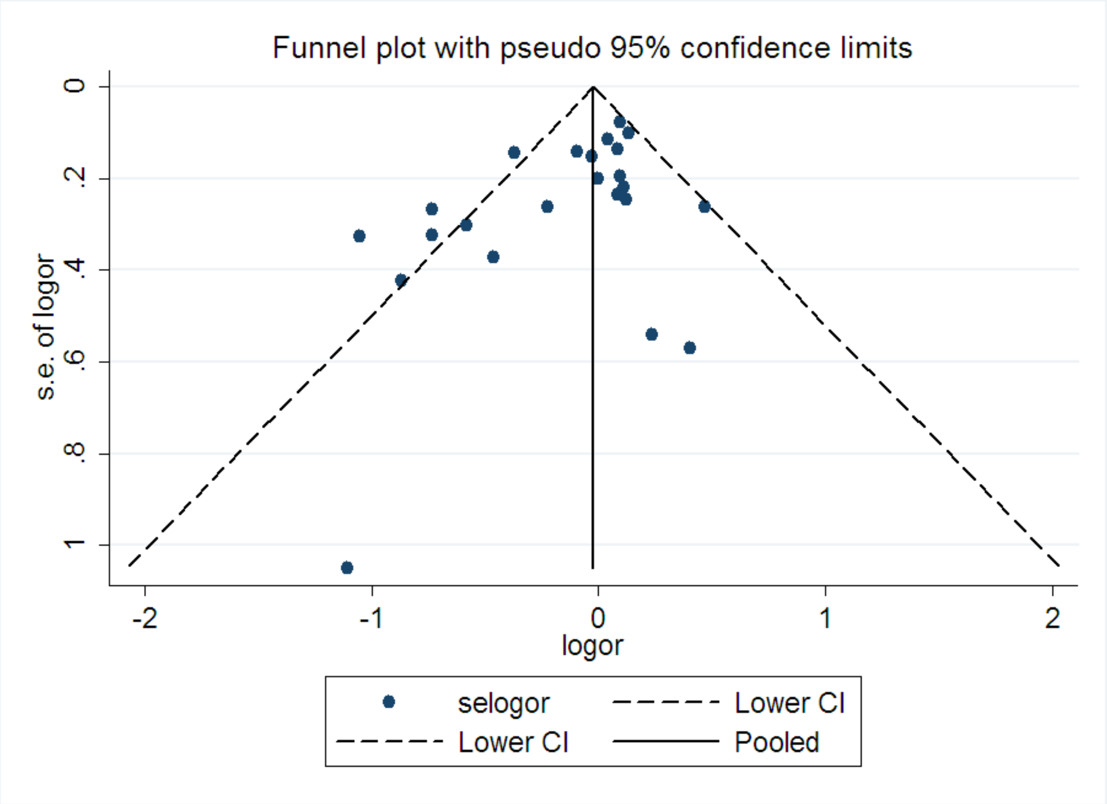

Supplementary Fig 4 Summary nonlinear dose-response curves: serum/plasma selenium and lung cancer

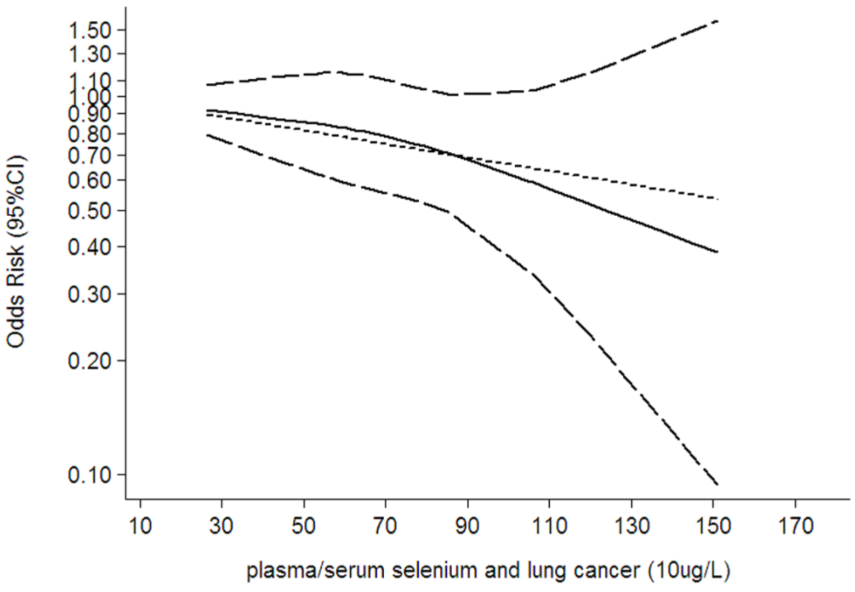

Supplementary Fig 5 Summary nonlinear dose-response curves: toenail selenium and bladder cancer

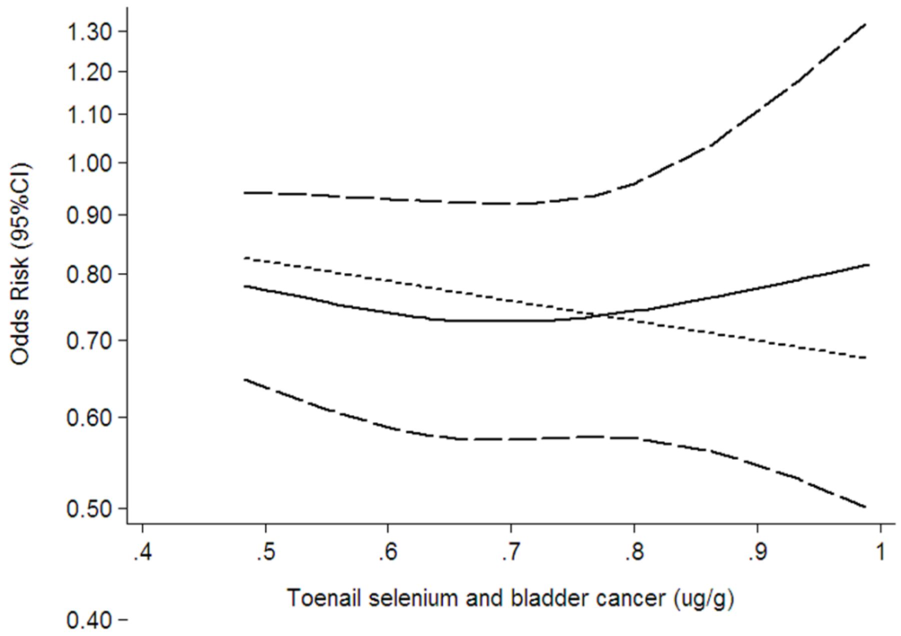

Supplementary Fig 6 Summary nonlinear dose-response curves: serum/plasma selenium and prostate cancer

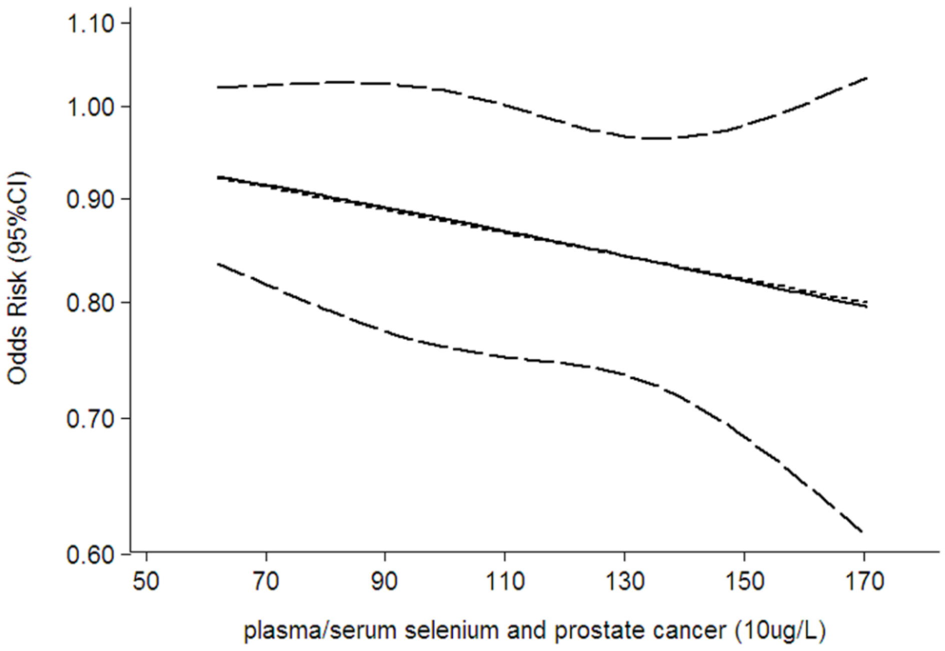

Supplementary Fig 7 Summary nonlinear dose-response curves: toenail selenium and prostate cancer

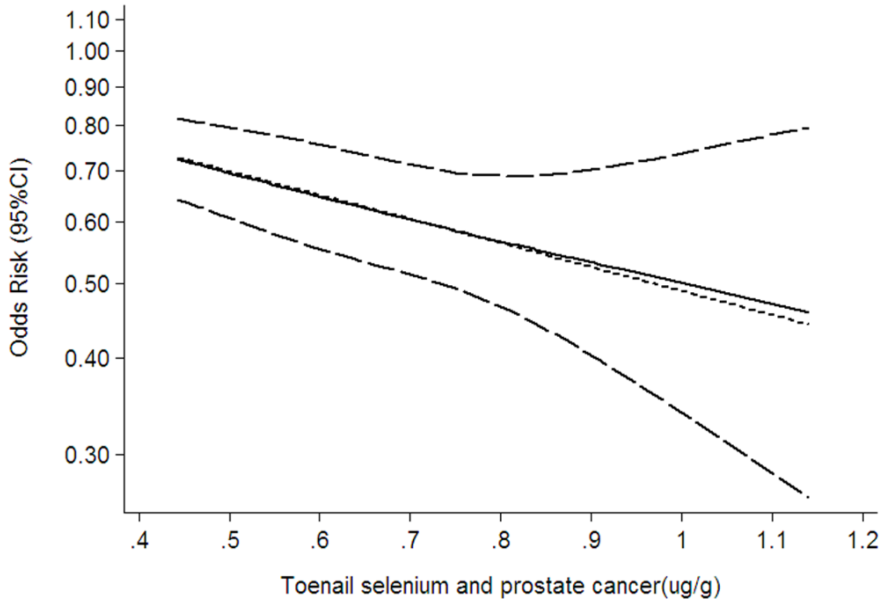

Supplement: supplementary table and figures [file srep19213-s1.pdf]
